# Algebraic Error Based Triangulation and Metric of Lines

**DOI:** 10.1371/journal.pone.0132354

**Published:** 2015-07-28

**Authors:** Fuchao Wu, Ming Zhang, Guanghui Wang, Zhanyi Hu

**Affiliations:** 1 National Laboratory of Pattern Recognition, Institute of Automation, Chinese Academy of Sciences, P.O. Box 2728, Beijing, 100190, China; 2 Department of Electrical Engineering & Computer Science, University of Kansas, 1520 West 15th Street, Lawrence, KS, 66045–7608, United States of America; Beijing University of Technology, CHINA

## Abstract

Line triangulation, a classical geometric problem in computer vision, is to determine the 3D coordinates of a line based on its 2D image projections from more than two views of cameras with known projection matrices. Compared to point features, line segments are more robust to matching errors, occlusions, and image uncertainties. In addition to line triangulation, a better metric is needed to evaluate 3D errors of line triangulation. In this paper, the line triangulation problem is investigated by using the Lagrange multipliers theory. The main contributions include: (i) Based on the Lagrange multipliers theory, a formula to compute the Plücker correction is provided, and from the formula, a new linear algorithm, LINa, is proposed for line triangulation; (ii) two optimal algorithms, OPTa-I and OPTa-II, are proposed by minimizing the algebraic error; and (iii) two metrics on 3D line space, the orthogonal metric and the quasi-Riemannian metric, are introduced for the evaluation of line triangulations. Extensive experiments on synthetic data and real images are carried out to validate and demonstrate the effectiveness of the proposed algorithms.

## Introduction

Line triangulation [1], [2] refers to the process of determining a 3D line given its projections in two or more images and the corresponding camera matrices. As one of the fundamental problems in computer vision, this problem is trivial in theory, since the corresponding 3D line is the intersection of the back-projection planes of the image lines. However, when the number of views is larger than 2, the back-projection planes usually do not intersect at one line in the 3D space due to measurement errors and image noise. This leads to find a 3D line that fits the measured data optimally, i.e., optimal line triangulation.

Minimizing the algebraic error of line triangulation is a linear least squares problem with a quadratic constraint (called the Klein constraint), as defined in Section 2 of this paper. Adrien and Sturm [3], [4] proposed a linear algorithm for the algebraic error minimization. This algorithm first finds a solution of the corresponding linear least squares problem (i.e., by ignoring the Klein constraint), then, the solution is corrected subsequently by a singular value decomposition (SVD) method with the Klein constraint enforcement. This algorithm yields only an approximation of the optimal solution to the algebraic error minimization. The paper [5] proposed a suboptimal solution to algebraic-error line triangulation. This algorithm finds a suboptimal solution of the original problem by relaxing the quadratic unit norm constraint to six linear constraints. However, this still cannot yield an optimal solution to algebraic error minimization. To the best of our knowledge, how to find optimal solution of the algebraic error minimization is still an open problem.

In studies on line triangulation, a natural question is that which one of the above three optimality criteria is the “best”? In order to answer this question, we need a criterion which is independent of the three optimality criteria to describe the “bestness”. One intuitive criterion is the 3D error, i.e. distance between a reconstructed line and its ground truth. The Euclidean distance does not give a reasonable measure since it is not an intrinsic distance on 3D line space. So far, no study on the metrics of 3D lines is available in the literature, and thus, it is still an open problem for the evaluation of line triangulations.

This paper focuses on the triangulations and metrics of lines. The main contributions are summarized as follows:

- Based on the Lagrange multipliers theory, a formula to compute the Plücker correction is given and this Plücker correction formula is used to establish a quasi-Riemannian metric in 3D line space. From the formula, a new linear algorithm, LINa, is proposed for line triangulation. The computational complexity of our new linear algorithm is much simpler compared with the SVD method in the literature.
- For the algebraic error minimization, two new algorithms, OPTa-I and OPTa-II, are proposed to find the optimal solution. The OPTa-I is based on finding roots of a system of 2-degree polynomial equations in five variables; and the OPTa-II is based on solving a system of polynomial equations in two variables (one polynomial is of 6-degree and the other is of 10-degree). The continuous homotopy algorithm [6], [7] is used to solve these systems of polynomial equations.
- Two new metrics on 3D line space, named as the orthogonal metric and the quasi-Riemannian metric, are proposed for the evaluation of line triangulations. The orthogonal metric is based on the angular distance on rotation groups [8] and the orthogonal representation of 3D lines [4]; and the quasi-Riemannian metric is based on the Riemannian metric on the 5-dimensional unit sphere and our proposed Plücker correction formula.

The rest of the paper is organized as follows. Section 2 presents some preliminaries used in the paper. The Plücker correction formula and a new linear algorithm are presented in Section 3. Section 4 elaborates the two optimal algorithms for the algebraic error minimization. Section 5 gives two new metrics on 3D line space. Some experimental results with synthetic and real data are presented in Section 6 and Section 7, respectively. Finally, the paper is concluded in Section 8.

## Preliminaries

### 2.1 Plücker Coordinates

In 3D projective space, the Plücker coordinates of a line is defined by a nonzero 6-vector:

$$L=\left(\begin{array}{c} x\times y\\ x_{4}y-y_{4}x \end{array}\right)≜\left(\begin{array}{c} u\\ v \end{array}\right)$$ (1)

where $X=\left(\begin{array}{c} x\\ x_{4} \end{array}\right),\;Y=\left(\begin{array}{c} y\\ y_{4} \end{array}\right)$ are two non-coincident points on the line. The Plücker coordinates is homogeneous since the two 6-vectors computed with two different pairs of points on the line are equal up to a nonzero factor. From Eq (1), it is easy to see that **u** ^T^ **v** = (x_4_ **y**−y_4_ **x**)^T^(**x**×**y**) = 0, i.e. the Plücker coordinates satisfies **u** ^T^ **v** = 0, or written in a matrix form:

$$L^{\text{T}}\text{K}L=0\;\left(\text{K}=\left(\begin{array}{cc} & {\text{I}}_{3}\\ {\text{I}}_{3} & \end{array}\right)\right)$$ (2)

In 5D projective space, the quadric defined by Eq (2) is called the Klein quadric [9], thus, the Plücker coordinates satisfies the Klein quadric constraint. Conversely, if a nonzero 6-vector satisfies the Klein constraint, it must be the Plücker coordinates of a line in a 3D projective space.

### 2.2 Point-Line Distance

In the image plane, the algebraic distance from a point **x** = (*x*,*y*,1)^T^ to a line **l** is defined as [10]:

$$\text{da}(x,l)=|x^{\text{T}}l|\;\left(l^{\text{T}}l\text{=1}\right)$$ (3)

Given a measured point set of a line **l**, *ℓ* = {**x**_*j*_ = (*x*_*j*_,*y*_*j*_,1)^*T*^: 1≤*j*≤*M*}, and let

$$l_{\text{a}}=\text{arg min}\sum_{j=1}^{M}{\text{da}}^{2}(x_{j},l)\text{ subject to }l^{\text{T}}l\text{=1}$$ (4)

then,**l**_a_ is called the linear least squares fitting of the measured point set *ℓ*, which has linear solution [10].

### 2.3 Optimality Criteria

Given *N* line-projection matrices,$P_{i}\left(1\leq i\leq N\right)$, and let *ℓ*_*i*_ = {**x**_*ij*_ = (*x*_*ij*_,*y*_*ij*_,1)^*T*^: 1≤*j*≤*M*_*i*_} be a measured point set from the imaged line $P_{i}L$ of a 3D line **L**, the line triangulation is meant to estimate the 3D line **L** from these measured point sets *ℓ*_*i*_(1≤*i*≤*N*). The algebraic distance of point-line in the image plane leads to the following optimality criteria to solve this problem [4], [10]:

$$\begin{array}{l} L_{\text{a}}^{*}=\text{arg min a}(L)≜\sum_{i=1}^{N}\sum_{j=1}^{M_{i}}{\text{da}}^{2}(x_{ij},P_{i}L)\\ \text{ subject to }L^{\text{T}}\text{K}L=0\text{ and }L^{\text{T}}L=1 \end{array}$$ (5)

where $L_{\text{a}}^{*}$ is called the optimal solution to minimize algebraic error. $L_{\text{a}}^{*}$ makes the sum of squared algebraic distances from the measured points **x**_*ij*_ to the re-projection lines $P_{i}L_{\text{a}}^{*}$ reach a minimum, thus, $\left\{P_{1}L_{\text{a}}^{*},P_{2}L_{\text{a}}^{*},\ldots ,P_{N}L_{\text{a}}^{*}\right\}$ are the linear least squares fittings of the measured point sets {*ℓ*_1_,*ℓ*_2_,…,*ℓ*_*N*_}.

The minimization term in Eq (5) can be expressed as

$$\begin{array}{l} \text{a}(L)=\sum_{i=1}^{N}\sum_{j=1}^{M_{i}}{\left(x_{ij}^{\text{T}}P_{i}L\right)}^{2}\\ \;\;\;\;\;\;\;\;=L^{\text{T}}\text{A}L\;\left(\text{where A}=\sum_{i=1}^{N}P_{i}^{\text{T}}\left(\sum_{j=1}^{M_{i}}x_{ij}^{}x_{ij}^{\text{T}}\right)P_{i}\right) \end{array}$$ (6)

Thus, the cost function Eq (5) can be rewritten as

$$\begin{array}{l} L_{\text{a}}^{*}=\text{arg min}L^{\text{T}}\text{A}L\\ \text{ subject to }L^{\text{T}}\text{K}L=0\text{ and }L^{\text{T}}L=1 \end{array}$$ (7)

which means that the minimization of the algebraic error is a linear least squares problem with the Klein constraint.

## Linear Solution to Minimize Algebraic Error

Adrien and Sturm [4] first proposed a linear algorithm to estimate $L_{\text{a}}^{*}$, which is divided into the following two steps:

(a) Solve the linear least squares problem:

$$\begin{array}{l} \bar{L}=\text{arg min}L^{\text{T}}\text{A}L\\ \text{ subject to }L^{\text{T}}L=1 \end{array}$$ (8)

The solution $\bar{L}$ is the eigenvector correspond to the matrix A’s smallest eigenvalue.

(b) Compute the nearest point $L_{\text{k}}^{*}$ from $\bar{L}$ to the Klein quadric as the final estimate:

$$\begin{array}{l} L_{\text{k}}^{*}=\text{arg min}\left\|L-\bar{L}\right\|\\ \text{ subject to }L^{\text{T}}\text{K}L=0 \end{array}$$ (9)

Adrien and Sturm [4] gave an SVD method to compute the nearest point $L_{\text{k}}^{*}$.

The step (b) in the above algorithm is called the Plücker correction. When there are errors in the measurement data, $\bar{L}$ does not strictly satisfy the Klein constraint, hence, it can not be the Plücker coordinates of a line in the 3D projective space. Thus, the Plücker correction is an important step in the algorithm. This section presents a formula to compute the Plücker correction and a new linear algorithm ‘LINa’.

### 3.1 Linear Algorithm LINa

We consider the following minimization:

$$\begin{array}{l} L_{\text{s}}^{*}=\text{arg min}\left\|L-\bar{L}\right\|\\ \text{ subject to }L^{\text{T}}\text{K}L=0\text{ and }L^{\text{T}}L=1 \end{array}$$ (10)

Although this minimization contains a unit norm constraint, it is in fact equivalent to Eq (9) according to the following Lemma.

**Lemma 1:** (a) If $L_{\text{s}}^{*}$ is the optimal solution of Eq (10), then $L_{\text{k}}≜L_{\text{s}}^{*\text{T}}\bar{L}\cdot L_{\text{s}}^{*}$ must be the optimal solution of Eq (9).

(b) Conversely, if $L_{\text{k}}^{*}$ is the optimal solution of Eq (9), then $L_{\text{s}}≜{\left(L_{\text{k}}^{*\text{T}}L_{\text{k}}^{*}\right)}^{-1/2}L_{\text{k}}^{*}$ must be the optimal solution of Eq (10).

**Proof:** For an arbitrary unit 6-vector **L**, there must be

$$\left\|L-\bar{L}\right\|=\sqrt{2}\left(1-L^{\text{T}}\bar{L}\right)$$ (11)

Since $L_{\text{s}}^{*}$ is the optimal solution of Eq (10), $0\leq L_{\text{s}}^{*\text{T}}\bar{L}\leq 1$ and

$$\left\|L_{\text{s}}^{*}-\bar{L}\right\|=\sqrt{2}(1-L_{\text{s}}^{*\text{T}}\bar{L})$$ (12)

Let $L_{\text{k}}=L_{\text{s}}^{*\text{T}}\bar{L}\cdot L_{\text{s}}^{*}$, then

$$\left\|L_{\text{k}}^{}-\bar{L}\right\|=\left\|L_{\text{s}}^{*\text{T}}\bar{L}\cdot L_{\text{s}}^{*}-\bar{L}\right\|=\sqrt{1-{\left(L_{\text{s}}^{*\text{T}}\bar{L}\right)}^{2}}$$ (13)

Let $L_{\text{s}}={\left(L_{\text{k}}^{*\text{T}}L_{\text{k}}^{*}\right)}^{-1/2}L_{\text{k}}^{*}$, then,

$$\left\|L_{\text{s}}^{}-\bar{L}\right\|=\sqrt{2}(1-L_{\text{s}}^{\text{T}}\bar{L})$$ (14)

Since $L_{\text{k}}^{*}$ is the optimal solution of Eq (9), $L_{\text{k}}^{*}=L_{\text{s}}^{\text{T}}\bar{L}\cdot L_{\text{s}}$ and $0\leq L_{\text{s}}^{\text{T}}\bar{L}\leq 1$, thus

$$\left\|L_{\text{k}}^{*}-\bar{L}\right\|=\left\|L_{\text{s}}^{\text{T}}L_{\text{k}}^{*}\cdot L_{\text{s}}-\bar{L}\right\|=\sqrt{1-{\left(L_{\text{s}}^{\text{T}}\bar{L}\right)}^{2}}$$ (15)

(a): If **L**_k_ is not the optimal solution of Eq (9), then,$\left\|L_{\text{k}}^{}-\bar{L}\right\|>\left\|L_{\text{k}}^{*}-\bar{L}\right\|$. From Eqs (13) and (15), we have

$$\sqrt{1-{\left(L_{\text{s}}^{*\text{T}}\bar{L}\right)}^{2}}>\sqrt{1-{\left(L_{\text{s}}^{\text{T}}\bar{L}\right)}^{2}}$$ (16)

and thus,$L_{\text{s}}^{*\text{T}}\bar{L}<L_{\text{s}}^{\text{T}}\bar{L}$. Then, by Eqs (12) and (14),$\left\|L_{\text{s}}^{*}-\bar{L}\right\|>\left\|L_{\text{s}}^{}-\bar{L}\right\|$, which is contrary to the fact that $L_{\text{s}}^{*}$ is the optimal solution of Eq (10). Therefore,**L**_k_ must be the optimal solution of Eq (9).

Similarly, (b) can be proved.

According to Eq (11), the minimization problem Eq (10) is simplified to

$$\begin{array}{l} L_{\text{s}}^{*}=\text{arg min}(1-L^{\text{T}}\bar{L})\\ \text{ subject to }L^{\text{T}}\text{K}L=0\text{ and }L^{\text{T}}L=1 \end{array}$$ (17)

Proposition 1 below gives an analytical expression of $L_{\text{s}}^{*}$. Compared with the SVD method to compute $L_{\text{k}}^{*}$, the computation of $L_{\text{s}}^{*}$ is much simpler.

**Proposition 1:** For $\bar{L}={\left({\bar{u}}^{\text{T}},\;{\bar{v}}^{\text{T}}\right)}^{\text{T}}\in R^{6}$ and $\left\|\bar{L}\right\|=1$, (a) The minimization Eq (10) has a unique solution if $\bar{u}\neq \pm \bar{v}$ as:

$$L_{\text{s}}^{*}=\frac{1}{2}\left(\begin{array}{c} \frac{\bar{u}+\bar{v}}{\left\|\bar{u}+\bar{v}\right\|}+\frac{\bar{u}-\bar{v}}{\left\|\bar{u}-\bar{v}\right\|}\\ \frac{\bar{u}+\bar{v}}{\left\|\bar{u}+\bar{v}\right\|}-\frac{\bar{u}-\bar{v}}{\left\|\bar{u}-\bar{v}\right\|} \end{array}\right)$$ (18a)

(b) The minimization Eq (10) has infinitely many solutions if $\bar{u}=\pm \bar{v}$ as:

$$L_{\text{s}}^{*}=\sqrt{2}\left(\begin{array}{l} {\bar{u}}^{\text{T}}d\cdot d\\ \pm (\bar{u}-{\bar{u}}^{\text{T}}d\cdot d) \end{array}\right)\;\left(d\in R^{3},\;\left\|d\right\|=\text{1}\right)$$ (18b)

**Fig 1:**
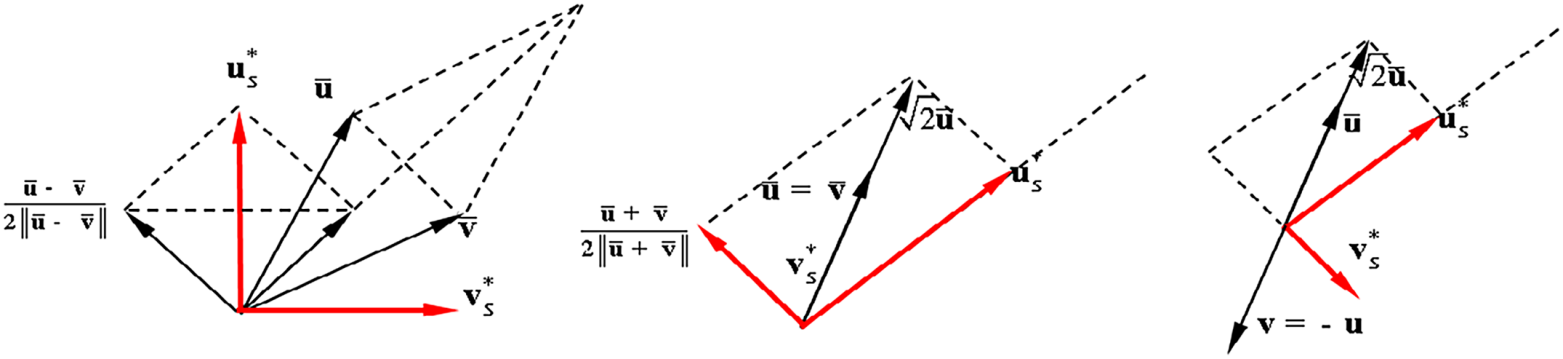
Geometric interpretation of the Plücker correction $L_{\text{s}}^{*}={\left(u_{\text{s}}^{*\text{T}},v_{\text{s}}^{*\text{T}}\right)}^{\text{T}}$.

The proof of the proposition is given in the next subsection. The geometric interpretations for Eqs (18a) and (18b) are shown in Fig 1. Since $\left\|\bar{u}+\bar{v}\right\|=\sqrt{1+2{\bar{u}}^{\text{T}}\bar{v}}$ and $\left\|\bar{u}-\bar{v}\right\|=\sqrt{1-2{\bar{u}}^{\text{T}}\bar{v}}$, Eq (18a) can be rewritten as

$$L_{\text{s}}^{*}=\frac{1}{2}\left(\begin{array}{c} \frac{\bar{u}+\bar{v}}{\sqrt{1+2{\bar{u}}^{\text{T}}\bar{v}}}+\frac{\bar{u}-\bar{v}}{\sqrt{1-2{\bar{u}}^{\text{T}}\bar{v}}}\\ \frac{\bar{u}+\bar{v}}{\sqrt{1+2{\bar{u}}^{\text{T}}\bar{v}}}-\frac{\bar{u}-\bar{v}}{\sqrt{1-2{\bar{u}}^{\text{T}}\bar{v}}} \end{array}\right)$$ (19)

Thus, when $\bar{L}$ satisfies the Klein constraint ${\bar{u}}^{\text{T}}\bar{v}=0$, there must be $L_{\text{s}}^{*}=\bar{L}$.

(a) $\bar{u}\neq \pm \bar{v}$(b) $\bar{u}=\bar{v}$(c) $\bar{u}=-\bar{v}$

Based on the above discussion, our linear algorithm LINa can be summarized in Table 1.

**Table 1:** Linear algorithm: LINa.

(a) Compute the eigenvector $\bar{L}$ associated with the smallest eigenvalue of matrix A
(b) Compute the Plücker correction $L_{\text{s}}^{*}$ by Eq (12) as the final estimation of $L_{\text{a}}^{*}$

**Remark 1:** In practice, the case (b) in Proposition 1 happens rarely. This is because the Klein constraint makes {**u**, **v**} orthogonal to each other, thus, they must be linearly independent of each other. When there are errors in the measurement data, the solution of Eq (8) cannot guarantee the orthogonality of {**u**, **v**}, except for their linear independency. Hence, the case (b) rarely happens in practice.

By Lemma 1 and Proposition 1, the optimal solution of Eq (15) can be obtained as:

(a) If $\bar{u}\neq \pm \bar{v}$, then

$$L_{\text{k}}^{*}=\frac{\left\|\bar{u}+\bar{v}\right\|+\left\|\bar{u}-\bar{v}\right\|}{4}\left(\begin{array}{c} \frac{\bar{u}+\bar{v}}{\left\|\bar{u}+\bar{v}\right\|}+\frac{\bar{u}-\bar{v}}{\left\|\bar{u}-\bar{v}\right\|}\\ \frac{\bar{u}+\bar{v}}{\left\|\bar{u}+\bar{v}\right\|}-\frac{\bar{u}-\bar{v}}{\left\|\bar{u}-\bar{v}\right\|} \end{array}\right)$$ (20a)

(b) If $\bar{u}=\pm \bar{v}$, then

$$L_{\text{k}}^{*}=\left(1+4{\left({\bar{u}}^{\text{T}}d\right)}^{2}\right)\left(\begin{array}{l} {\bar{u}}^{\text{T}}d\cdot d\\ \pm (\bar{u}-{\bar{u}}^{\text{T}}d\cdot d) \end{array}\right)\;\left(d^{\text{T}}d=1\right)$$ (20b)

### 3.2 Proof of Proposition 1

Construct the Lagrange function of Eq (17) as follows:

$$f(L,\alpha ,\beta )=(1-L^{\text{T}}\bar{L})-\alpha (\;L^{\text{T}}\text{K}L)-\beta (L^{\text{T}}L-1)$$ (21)

According to the optimization theory [11], the solution of Eq (17) must be a stationary point of the Lagrange function, i.e., there are multipliers (α*,β*) such that $\left(L_{\text{s}}^{*},\alpha^{*},\beta^{*}\right)$ is a solution of the following Lagrange equations:

$$\{\begin{array}{l} \frac{\partial f}{\partial L}=-2(\alpha \text{K}+\beta \text{I})L-\bar{L}=0\\ \frac{\partial f}{\partial \alpha }=L^{\text{T}}\text{K}L=0\\ \frac{\partial f}{\partial \beta }=L^{\text{T}}L-1=0 \end{array}$$ (22)

Thus, by solving the Lagrange equations we can obtain the optimal solution $L_{\text{s}}^{*}$. The first equation in Eq (25) can be rewritten as

$$\{\begin{array}{l} (\alpha +\beta )(u+v)=-\frac{\bar{u}+\bar{v}}{2}\\ (\alpha -\beta )(u-v)=-\frac{\bar{v}-\bar{u}}{2} \end{array}$$ (23)

From the last two equations in Eq (22), we have

u≠±v (24)

(i) If $\bar{u}\neq \pm \bar{v}$, then by Eqs (23) and (24), we have

α+β≠0, α−β≠0 (25)

Let *α*′ = (*α*+*β*)^−1^ and *β*′ = (*α*−*β*)^−1^, then from Eq (23) we have

$$L=-\frac{1}{4}\left(\begin{array}{c} \alpha^{\prime }(\bar{u}+\bar{v})-\beta^{\prime }(\bar{u}-\bar{v})\\ \alpha^{\prime }(\bar{u}+\bar{v})+\beta^{\prime }(\bar{u}-\bar{v}) \end{array}\right)=-\frac{1}{4}\left(\begin{array}{c} \alpha^{\prime }a-\beta^{\prime }b\\ \alpha^{\prime }a+\beta^{\prime }b \end{array}\right)\;\left(\text{where }\left(\begin{array}{c} a\\ b \end{array}\right)=\left(\begin{array}{c} \bar{u}+\bar{v}\\ \bar{u}-\bar{v} \end{array}\right)\right)$$ (26)

Thus,

$$\begin{array}{l} L^{\text{T}}\text{K}L=0\;\Leftrightarrow \;{\left(\alpha^{\prime }a-\beta^{\prime }b\right)}^{\text{T}}(\alpha^{\prime }a+\beta^{\prime }b)=0\\ \;\;\;\;\;\;\;\;\;\;\;\;\;\;\;\;\Leftrightarrow \;{\alpha^{\prime }}^{2}a^{\text{T}}a-{\beta^{\prime }}^{2}b^{\text{T}}b=0 \end{array}$$ (27)

$$\begin{array}{l} L^{\text{T}}L=1\;\Leftrightarrow \;{\left(\alpha^{\prime }a-\beta^{\prime }b\right)}^{\text{T}}(\alpha^{\prime }a-\beta^{\prime }b)+{\left(\alpha^{\prime }a+\beta^{\prime }b\right)}^{\text{T}}(\alpha^{\prime }a+\beta^{\prime }b)=16\\ \;\;\;\;\;\;\;\;\;\;\;\;\Leftrightarrow \;{\alpha^{\prime }}^{2}a^{\text{T}}a+{\beta^{\prime }}^{2}b^{\text{T}}b=8 \end{array}$$ (28)

Therefore, the following linear equations on (*α*′2,*β*′2) hold:

$$\left(\begin{array}{cc} a^{\text{T}}a & -b^{\text{T}}b\\ a^{\text{T}}a & b^{\text{T}}b \end{array}\right)\left(\begin{array}{c} {\alpha^{\prime }}^{2}\\ {\beta^{\prime }}^{2} \end{array}\right)=\left(\begin{array}{c} 0\\ 8 \end{array}\right)$$ (29)

then,

$$\{\begin{array}{c} \alpha^{\prime }=\frac{2}{\pm \sqrt{a^{\text{T}}a}}\\ \beta^{\prime }=\frac{2}{\pm \sqrt{b^{\text{T}}b}} \end{array}$$ (30)

Substituting Eq (30) into Eq (26) gives the following four solutions to **L**:

$$L_{\pm ,\pm }=\pm \frac{1}{2\sqrt{b^{\text{T}}b}}\left(\begin{array}{c} b\\ -b \end{array}\right)\pm \frac{1}{2\sqrt{a^{\text{T}}a}}\left(\begin{array}{c} a\\ a \end{array}\right)=\pm \frac{1}{2\left\|\bar{u}-\bar{v}\right\|}\left(\begin{array}{c} \bar{u}-\bar{v}\\ -(\bar{u}-\bar{v}) \end{array}\right)\pm \frac{1}{2\left\|\bar{u}+\bar{v}\right\|}\left(\begin{array}{c} \bar{u}+\bar{v}\\ \bar{u}+\bar{v} \end{array}\right)$$ (31)

**Fig 2:**
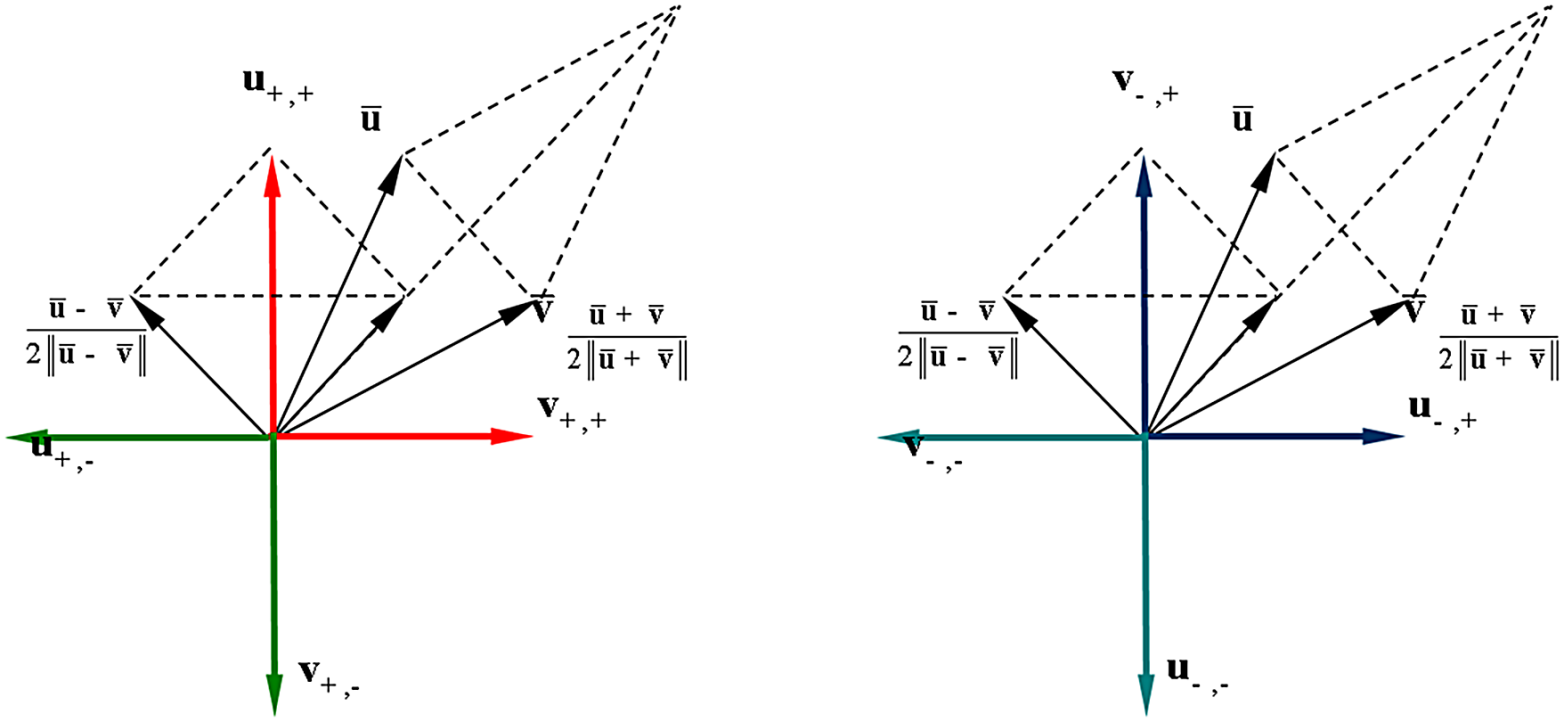
Geometric interpretation for $L_{\pm ,\pm }={\left(u_{\pm ,\pm }^{\text{T}},v_{\pm ,\pm }^{\text{T}}\right)}^{\text{T}}$.

The geometric interpretations of the four solutions are shown in Fig 2.

It can be easily verified that $L_{+,+}=\text{arg min}\{1-L_{\pm ,\pm }^{\text{T}}{\bar{L}}_{\pm ,\pm }\}$, and thus,

$$L_{s}^{*}=L_{+,+}=\frac{1}{2}\left(\begin{array}{c} \frac{\bar{u}+\bar{v}}{\left\|\bar{u}+\bar{v}\right\|}+\frac{\bar{u}-\bar{v}}{\left\|\bar{u}-\bar{v}\right\|}\\ \frac{\bar{u}+\bar{v}}{\left\|\bar{u}+\bar{v}\right\|}-\frac{\bar{u}-\bar{v}}{\left\|\bar{u}-\bar{v}\right\|} \end{array}\right)$$ (32)

(ii) When $\bar{u}=\bar{v}$, there must be ${\bar{u}}^{\text{T}}\bar{u}=\frac{1}{2}({\bar{u}}^{\text{T}}\bar{u}+{\bar{v}}^{\text{T}}\bar{v})=\frac{1}{2}$. According to Eq (24) and the second equation of Eq (23), we have *β* = *α*. Substituting it into the first equation in Eq (23), we have

$$2\alpha (u+v)=-\frac{\bar{u}+\bar{v}}{2}=-\bar{u}$$ (33)

Therefore,

$$\alpha^{2}{\left(u+v\right)}^{\text{T}}(u+v)={\bar{u}}^{\text{T}}\bar{u}=\frac{1}{2}$$ (34)

By the last two equations in Eq (22), we have

$${\left(u+v\right)}^{\text{T}}(u+v)=u^{\text{T}}u+2u^{\text{T}}v^{\text{T}}+v^{\text{T}}v=1$$ (35)

Thus,$\alpha =\pm \frac{1}{\sqrt{2}}$. Substituting it into Eq (23), we have

$$v=-u-\sqrt{2}\bar{u}\;\text{or}\;v=-u+\sqrt{2}\bar{u}$$ (36)

If $v=-u-\sqrt{2}\bar{u}$, then

$$L^{\text{T}}\text{K}L=0\;\Leftrightarrow \;u^{\text{T}}u+\sqrt{2}u^{\text{T}}\bar{u}=0$$ (37)

$$L^{\text{T}}L-1=0\;\Leftrightarrow \;u^{\text{T}}u+{\left(u+\sqrt{2}\bar{u}\right)}^{\text{T}}\left(u+\sqrt{2}\bar{u}\right)=1\Leftrightarrow \;u^{\text{T}}u+\sqrt{2}u^{\text{T}}\bar{u}=0$$ (38)

Thus,

$$\;u^{\text{T}}u+\sqrt{2}u^{\text{T}}\bar{u}=0\Leftrightarrow \{\begin{array}{c} L^{\text{T}}\text{K}L=0\;\\ L^{\text{T}}L-1=0 \end{array}$$ (39)

and $\forall u\in S_{-}≜\left\{u:u^{\text{T}}u+\sqrt{2}u^{\text{T}}\bar{u}=0\right\}$,

$$1-L^{\text{T}}\bar{L}=1-u^{\text{T}}\bar{u}-{\left(-u-\sqrt{2}\bar{u}\right)}^{\text{T}}\bar{u}=\;1+\sqrt{2}{\bar{u}}^{\text{T}}\bar{u}=1+\frac{\sqrt{2}}{2}$$ (40)

Similarly, if $v=-u+\sqrt{2}\bar{u}$, then

$$u^{\text{T}}u-\sqrt{2}u^{\text{T}}\bar{u}=0\Leftrightarrow \{\begin{array}{c} L^{\text{T}}\text{K}L=0\;\\ L^{\text{T}}L-1=0 \end{array}$$ (41)

and $\forall u\in S_{+}≜\left\{u:u^{\text{T}}u-\sqrt{2}u^{\text{T}}\bar{u}=0\right\}$,

$$1-L^{\text{T}}\bar{L}=1-\frac{\sqrt{2}}{2}$$ (42)

By Eqs (40) and (42), $L_{s}^{*}$ has infinitely many solutions:

$$L_{s}^{*}=\left(\begin{array}{c} u\\ -u+\sqrt{2}\bar{u} \end{array}\right),\;u\in S_{+}$$ (43)

Next, we consider the set $S_{+}$. Let **u** = *s* **d** (where **d** is a unit 3-vector, *s*≠0), then

$$u^{\text{T}}u-\sqrt{2}u^{\text{T}}\bar{u}=0\Leftrightarrow s^{\text{2}}-s\sqrt{2}d^{\text{T}}\bar{u}=0\Leftrightarrow s=\sqrt{2}{\bar{u}}^{\text{T}}d$$ (44)

thus, $S_{+}=\left\{u=\sqrt{2}{\bar{u}}^{\text{T}}d\cdot d\;:\;d^{\text{T}}d=1\right\}$. Therefore Eq (43) can be rewritten as

$$L_{\text{s}}^{*}=\sqrt{2}\left(\begin{array}{l} {\bar{u}}^{\text{T}}d\cdot d\\ {\bar{u}}^{\text{T}}d\cdot d-\bar{u} \end{array}\right)\;\left(d^{\text{T}}d=1\right)$$ (45)

(iii) Similarly, when $\bar{u}=-\bar{v}$, $L_{s}^{*}$ also has infinitely many solutions:

$$L_{\text{s}}^{*}=\sqrt{2}\left(\begin{array}{l} {\bar{u}}^{\text{T}}d\cdot d\\ \bar{u}-{\bar{u}}^{\text{T}}d\cdot d \end{array}\right)\;\left(d^{\text{T}}d=1\right).$$ (46)

## Optimal Solution by Minimizing Algebraic Error

The algorithm LINa can only provide an approximate solution by minimizing algebraic errors. This section will present two algorithms ‘OPTa-I’ and ‘OPTa-II’ to compute the optimal solution. The algorithm OPTa-I converts the optimization problem to that of finding the real solutions of two systems of 2-degree polynomial equations in five variables, and the algorithm OPTa-II to that of finding the real solutions of a system of polynomial equations in two variables (one is of 6-degree, and the other is of 10-degree).

### 4.1 Algorithm OPTa-I

The optimal algorithm OPTa-I is summarized in Table 2.

**Table 2:** The optimal algorithm: OPTa-I.

(a) Construct the following system of polynomial equations by the matrix: A (see Eq (6)):
$$\{\begin{array}{l} u^{\text{T}}v=0\\ u^{\text{T}}u+v^{\text{T}}v=1\\ {\left[u+v\right]}_{\times }\left(({\text{A}}_{11}+{\text{A}}_{21})u+({\text{A}}_{12}+{\text{A}}_{22})v\right)=0\\ {\left[u-v\right]}_{\times }\left(({\text{A}}_{11}-{\text{A}}_{21})u+({\text{A}}_{12}-{\text{A}}_{22})v\right)=0 \end{array}\;\left(\text{where A}=\left(\begin{array}{cc} {\text{A}}_{11} & {\text{A}}_{12}\\ {\text{A}}_{21} & {\text{A}}_{22} \end{array}\right)\right)$$ (47)
Then compute its real solution set $S^{\text{I}}$;
(b) Determine the optimal solution by
$$L_{a}^{*}=\text{arg min}\left\{L^{\text{T}}\text{A}L\;:\;L=\left(\begin{array}{c} u\\ v \end{array}\right)\in S^{\text{I}}\right\}$$ (48)

Eq (47) is a system of 2-degree polynomial equations in six variables, and it has at most 64 real solutions based on the algebraic equations theory. Proposition 2 next shows this system can be simplified into two systems of 2-degree polynomial equations in five variables. Here we at first prove that Eq (48) is the optimal solution to the algebraic error minimization.

**Proof:** Consider the Lagrange function and the Lagrange equations of Eq (7):

$$f_{a}(L,\alpha ,\beta )=L^{\text{T}}\text{A}L-\alpha (\;L^{\text{T}}\text{K}L)-\beta (L^{\text{T}}L-1)$$ (49)

$$\{\begin{array}{l} \frac{\partial f_{a}}{\partial L}=2\left(\text{A}-\alpha \text{K}-\beta \text{I})\right)L=0\\ \frac{\partial f_{a}}{\partial \alpha }=L^{\text{T}}\text{K}L=0\;\\ \frac{\partial f_{a}}{\partial \beta }=L^{\text{T}}L-1\;=0 \end{array}$$ (50)

The first equation in Eq (50) can be rewritten as

$$\;\{\begin{array}{c} {\text{A}}_{11}u+{\text{A}}_{12}v=\alpha v+\beta u\\ {\text{A}}_{21}u+{\text{A}}_{22}v=\alpha u+\beta v \end{array}$$ (51)

It is obvious that this equation is equivalent to the following equation:

$$\;\{\begin{array}{c} \left({\text{A}}_{11}+{\text{A}}_{21})u+({\text{A}}_{12}+{\text{A}}_{22})v=(\alpha +\beta )(u+v\right)\\ \left({\text{A}}_{11}-{\text{A}}_{21})u+({\text{A}}_{12}-{\text{A}}_{22})v=(\beta -\alpha )(u-v\right) \end{array}$$ (52)

By eliminating the multipliers (*α*,*β*) in the above equation, we obtain the following 2-degree polynomial equations in (**u**,**v**):

$$\{\begin{array}{c} {\left[u+v\right]}_{\times }\left(({\text{A}}_{11}+{\text{A}}_{21})u+({\text{A}}_{12}+{\text{A}}_{22})v\right)=0\\ {\left[u-v\right]}_{\times }\left(({\text{A}}_{11}-{\text{A}}_{21})u+({\text{A}}_{12}-{\text{A}}_{22})v\right)=0 \end{array}$$ (53)

The last two equations in Eq (50) can be rewritten as

$$\{\begin{array}{l} u^{\text{T}}v=0\\ u^{\text{T}}u+v^{\text{T}}v=1 \end{array}$$ (54)

By combining Eqs (53) and (54), we have Eq (47). **L** from the stationary points (**L**,*α*,*β*) of the Lagrange function Eq (49) must be a real solution of Eq (47), thus the optimal solution of Eq (7) must belong to the real solution set of Eq (47), i.e.,$L_{\text{a}}^{*}\in S^{\text{I}}$. Therefore,

$$L_{\text{a}}^{*}=\text{arg min}\left\{L^{\text{T}}\text{A}L\;:\;L\in S^{\text{I}}\right\}$$ (55)

**Proposition 2:** The solution set of Eq (47) is the union of solution sets of two systems of 2-degree polynomial equations in five variables.

**Proof:** Let S be the solution set of Eq (47), then it must be the union of the following two sets:

$$\begin{array}{l} S_{0}=\left\{L={\left(u^{\text{T}},v^{\text{T}}\right)}^{\text{T}}\in S\;:\;v_{3}=0\right\}\\ S_{1}=\left\{L={\left(u^{\text{T}},v^{\text{T}}\right)}^{\text{T}}\in S\;:\;v_{3}\neq 0\right\} \end{array}$$ (56)

Clearly,$S_{0}$ is the solution set of the system of 2-degree polynomial equations in five variables obtained by setting *v*_3_ = 0 in Eq (47). For the set $S_{1}$, we consider the resulting equation system obtained by removing the unit norm constraint in Eq (47):

$$\{\begin{array}{l} u^{\text{T}}v=0\\ {\left[u+v\right]}_{\times }\left(({\text{A}}_{11}+{\text{A}}_{21})u+({\text{A}}_{12}+{\text{A}}_{22})v\right)=0\\ {\left[u-v\right]}_{\times }\left(({\text{A}}_{11}-{\text{A}}_{21})u+({\text{A}}_{12}-{\text{A}}_{22})v\right)=0 \end{array}\;$$ (57)

It is second order homogeneous on **L** = (**u** ^T^,**v** ^T^)^T^, and the set formed by normalizations of its all nonzero solutions is just the solution set S of Eq (47). Thus, let ${\tilde{S}}_{1}$ be the solution set of the system of 2-degree polynomial equations in five variables obtained by setting *v*_3_ = 1 in Eq (57), there must be

$$S_{1}=\left\{L=\frac{{\left({\tilde{u}}^{\text{T}},\;{\tilde{v}}^{\text{T}},1\right)}^{\text{T}}}{\sqrt{{\tilde{u}}^{\text{T}}\tilde{u}+{\tilde{v}}^{\text{T}}\tilde{v}+1}}\;:\;\left(\begin{array}{c} \tilde{u}\\ \tilde{v} \end{array}\right)\in {\tilde{S}}_{1}\right\}$$ (58)

Hence, Proposition 2 holds.

### 4.2 Algorithm OPTa-II

Let A(*α*,*β*) = A−*α*K−*β*I and its adjoint matrix be denoted as ${\text{A}}^{*}\text{(}\alpha ,\beta \text{)=(}A_{1}^{*}\text{(}\alpha ,\beta \text{),}A_{2}^{*}\text{(}\alpha ,\beta \text{),}\ldots \text{,}A_{6}^{*}\text{(}\alpha ,\beta \text{))}$, all its elements are at most 5-degree polynomials of (*α*,*β*). It is easy to see,

$$A_{}^{i\text{T}}\text{(}\alpha ,\beta \text{)}A_{j}^{\text{*}}\text{(}\alpha ,\beta \text{)}=\{\begin{array}{l} \text{det}(\text{A(}\alpha ,\beta \text{)}),\;i=j\\ 0,\;i\neq j \end{array}$$ (59)

where **A** ^iT^(*α*,*β*) is the *i*-th row vector of A(*α*,*β*), therefore,

$$\text{A(}\alpha ,\beta {\text{)A}}^{*}\text{(}\alpha ,\beta \text{)}=\text{det}(\text{A(}\alpha ,\beta \text{)})\text{I}$$ (60)

For each *k* (1≤*k*≤6),$A_{k}^{*}\text{(}\alpha ,\beta \text{)}=\text{0}$ is a system of 5-degree polynomial equations of (*α*,*β*), whose real solution set is denoted as $S_{k}=\{(\alpha_{i}^{k},\beta_{i}^{k}):0\leq i\leq t_{k}\}$. Next, we prove that this system has at least one real solution, i.e.$S_{k}\neq \emptyset$.

Let ${\text{A}}^{d(k)}(\alpha ,\beta )=\left(a_{1}(\alpha ,\beta ),a_{2}(\alpha ,\beta ),\ldots ,a_{6}(\alpha ,\beta )\right)$ be the sub-matrix formed by deleting the k-th row of A(*α*,*β*), then $A_{k}^{*}(\alpha ,\beta )=0$ can be expressed as

(a):

$$\{\begin{array}{c} \text{det}\left(a_{2}(\alpha ,\beta ),a_{3}(\alpha ,\beta ),\ldots ,a_{6}(\alpha ,\beta )\right)=0\\ \text{det}\left(a_{1}(\alpha ,\beta ),a_{3}(\alpha ,\beta ),\ldots ,a_{6}(\alpha ,\beta )\right)=0\\ \vdots \\ \text{det}\left(a_{1}(\alpha ,\beta ),a_{2}(\alpha ,\beta ),\ldots ,a_{5}(\alpha ,\beta )\right)=0 \end{array}$$ (61)

and it has the same solutions as the following equation system:

(b):

$$\{\begin{array}{c} \text{det}\left(a_{2}(\alpha ,\beta ),a_{3}(\alpha ,\beta ),\ldots ,a_{6}(\alpha ,\beta )\right)=0\\ \text{det}\left(a_{1}(\alpha ,\beta ),a_{3}(\alpha ,\beta ),\ldots ,a_{6}(\alpha ,\beta )\right)=0 \end{array}$$ (62)

This is because: From (b), both **a**_1_ and **a**_2_ can be linearly represented with $A≜\{a_{3},\;a_{4},a_{5},\;a_{6}\}$, thus, for arbitrary $a_{i},\;a_{j},a_{k}\in A$, {**a**_1_, **a**_2_,**a**_*i*_, **a**_*j*_,**a**_*k*_} must be linearly dependent, i.e., det(**a**_1_, **a**_2_,**a**_*i*_, **a**_*j*_,**a**_*k*_) = 0. Hence, solutions of (b) must be the ones of (a). Obviously, solutions of (a) are also the ones of (b). Therefore, (a) has the same solutions with (b).

Since non-real solutions of a system of real polynomial equations occur in complex conjugate pairs, there is at least one real solution in the 25 solutions of (b). Thus, $A_{k}^{*}(\alpha ,\beta )=0$ has at least one real solution.

The algorithm OPTa-II is summarized in Table 3.

**Table 3:** The optimal algorithm: OPTa-II.

(a) Compute the adjoint matrix, ${\text{A}}^{*}\text{(}\alpha ,\beta \text{)=(}A_{1}^{*}\text{(}\alpha ,\beta \text{),}A_{2}^{*}\text{(}\alpha ,\beta \text{),}\ldots \text{,}A_{6}^{*}\text{(}\alpha ,\beta \text{))}$, and choose *k*_1_,*k*_2_ such that the following system of polynomial equations has no real solutions:
$$\{\begin{array}{c} A_{k_{1}}^{*}\text{(}\alpha ,\beta \text{)}=\text{0}\\ A_{k_{2}}^{*}\text{(}\alpha ,\beta \text{)}=\text{0} \end{array}$$ (63)
(b) Compute the real solution set $S_{i}^{\text{II}}(i=1,2)$ of the following system of polynomial equations:
$$\{\begin{array}{l} \text{det(A(}\alpha ,\beta \text{)})=0\\ A_{k_{i}}^{*\text{T}}\text{(}\alpha ,\beta \text{)K}A_{k_{i}}^{*}\text{(}\alpha ,\beta \text{)}=\text{0} \end{array}$$ (64)
(c) Determine the optimal solution from:
$$L_{\text{a}}^{*}=\text{arg min}\left\{L_{i}^{\text{T}}\text{(}\alpha ,\beta \text{)A}L_{i}\text{(}\alpha ,\beta \text{) }:\;L_{i}\text{(}\alpha ,\beta \text{)}=\frac{A_{k_{i}}^{*}\text{(}\alpha ,\beta \text{)}}{\left\|A_{k_{i}}^{*}\text{(}\alpha ,\beta \text{)}\right\|},\;(\alpha ,\beta )\in S_{i}^{\text{II}},\;i=1,2\right\}$$ (65)

For the two polynomial equations in the step (b) of OPTa-II, one is of 6-degree and the other is of 10-degree, and thus it has at most 60 real solutions based on the algebraic equations theory. Next, we prove that Eq (65) is the optimal solution to the algebraic error minimization.

**Proof:** The first equation in Eq (50) can be rewritten as A(*α*,*β*)**L** = 0, thus, **L**≠0 leads to the following 6-degree polynomial equation of (*α*,*β*):

det(A(α,β))=0 (66)

i.e., the multipliers (*α*,*β*) of the stationary points (**L**,*α*,*β*) of the Lagrange function Eq (48) satisfies Eq (66).

Since the system of polynomial equations $\left\{A_{k_{1}}^{*}\text{(}\alpha ,\beta \text{)}=\text{0},\;A_{k_{2}}^{*}\text{(}\alpha ,\beta \text{)}=\text{0}\right\}$ has no real solutions, there must be $A_{k_{1}}^{*}\text{(}\alpha ,\beta \text{)}\neq \text{0}$ or $A_{k_{2}}^{*}\text{(}\alpha ,\beta \text{)}\neq \text{0}$ for (*α*,*β*)∈*R* ^2^. This leads to rank(A(*α*,*β*)) = 5 for (*α*,*β*)∈*R* ^2^. Therefore, from Eqs (60) and (66), **L** of the stationary points (**L**,*α*,*β*) can be expressed as

$$L_{1}(\alpha ,\beta )=s_{1}A_{k_{1}}^{*}\text{(}\alpha ,\beta \text{) or }L_{2}(\alpha ,\beta )=s_{2}A_{k_{2}}^{*}\text{(}\alpha ,\beta \text{) }(s_{1}\neq 0,s_{2}\neq 0)$$ (67)

By the second equation in Eq (50), the multipliers (*α*,*β*) of the stationary points (**L**,*α*,*β*) must belong to one of the real solution sets of the following two systems of polynomial equations:

$$\left\{ \begin{array}{l} \text{det(A(}\alpha ,\beta \text{)})=0\\ A_{k_{i}}^{*\text{T}}\text{(}\alpha ,\beta \text{)K}A_{k_{i}}^{*}\text{(}\alpha ,\beta \text{)}=\text{0} \end{array}.\left(i=1,2\right)\right.$$ (68)

i.e.,$(\alpha ,\beta )\in S_{1}^{\text{II}}\cup S_{2}^{\text{II}}$. Thus, from Eq (66) and the unit norm constraint **L** ^T^ **L** = 1, **L** of the stationary points (**L**,*α*,*β*) must belong to the following set:

$$S=\left\{L_{i}(\alpha ,\beta )=\frac{A_{k_{i}}^{\text{*}}(\alpha ,\beta )}{\left\|A_{k_{i}}^{\text{*}}(\alpha ,\beta )\right\|}\;:\;(\alpha ,\beta )\in S_{i}^{\text{II}},i=1,2\right\}$$ (69)

Therefore,

$$\begin{array}{l} L_{\text{a}}^{*}=\text{arg min}\left\{L^{\text{T}}\text{A}L\;:\;L\in S\right\}\\ \;\;\;\;\;=\text{arg min}\left\{L_{i}^{\text{T}}\text{(}\alpha ,\beta \text{)A}L_{i}\text{(}\alpha ,\beta \text{) }:\;L_{i}\text{(}\alpha ,\beta \text{)}=\frac{A_{k_{i}}^{*}\text{(}\alpha ,\beta \text{)}}{\left\|A_{k_{i}}^{*}\text{(}\alpha ,\beta \text{)}\right\|},\;(\alpha ,\beta )\in S_{i}^{\text{II}},\;i=1,2\right\} \end{array}$$ (70)

The algorithm OPTa-II needs only to solve some systems of polynomial equations in two variables, it effectively simplifies the algorithm OPTa-I. If for any {*i*,*j*} pairs, the system of polynomial equations $\{A_{i}^{*}\text{(}\alpha ,\beta \text{)}=\text{0},$ $A_{j}^{*}\text{(}\alpha ,\beta \text{)}=\text{0\}}$ has real solution, the algorithm OPTa-II may fail. However, this situation never happened in our extensive numerical simulations.

**Remark 2:** In the experiments of this paper, we use the continuous homotopy method [6] [7] to solve the system of polynomial equations. The method is first proposed in [12]. Through 30 years of efforts of many researchers, the method has made a great success in computing zero points of non-linear mappings. It can give all zero points of a polynomial mapping [6][7][13]. In the field of computer vision, the method has been used to solve self-calibrations of cameras, such as the Kruppa equations [14], the modulus constraint equations and the absolute quadric constraint equations [15]. For the 2-degree polynomials with five variables in OPTa-I and the high-degree polynomials with two variables in OPTa-II, the continuous homotopy method is of high computational efficiency.

## Metrics on 3D Line Space

In order to evaluate 3D errors of line triangulations, we need a metric in 3D line space. The Euclidean distance

$d_{\text{E}}(L,\;L^{\prime })≜\text{min}\left\{\left\|L-L^{\prime }\right\|,\;\left\|L-(-L^{\prime })\right\|\right\}$(where **L**, **L**′ are the normalized Plücker coordinates of lines $L,\;L^{\prime }$) is not appropriate for the evaluation of line triangulations since is not an intrinsic distance on 3D line space. The aim of this section is to introduce two new metrics on 3D line space, called the orthogonal metric and the quasi-Riemannian metric. Compared with the Euclidean metric and the orthogonal metric, the quasi-Riemannian metric appears more appropriate.

In this section, the 5-dimensional unit sphere centered at the origin in $R^{6}$ is denoted by $S^{5}(1)$, and the intersection of the Klein quadric K and $S^{5}(1)$ is denoted by $K(1)≜S^{5}(1)\cap K$, which is a 4-dimensional smooth sub-manifold of $S^{5}(1)$, called the unit Klein quadric.

### 5.1 Orthogonal Metric in 3D Line Space

The proposed orthogonal metric is mainly from the angular distance of rotation matrices [8] and the orthogonal representation of 3D lines [4]. The angular metric on rotation group is given in Appendix I.

If $L=\left(\begin{array}{c} u\\ v \end{array}\right)\in K(1)$, then (a): **u**≠0,**v**≠0; or (b):**u** = 0,‖**v**‖ = 1; or (c): ‖**u**‖ = 1,**v** = 0. By the definition of Plücker coordinates, for the case (b), **L** is the Plücker coordinates of a 3D line passing through the origin and **v** is its direction; for the case (c), **L** is the Plücker coordinates of a 3D line on the infinite plane and **u** is its normalized coordinates as a 2D line on the infinite plane.

Let $K_{a}(1)=\left\{L\in K(1):u\neq 0,v\neq 0\right\}$,$K_{b}(1)=\left\{L\in K(1):u=0,\left\|v\right\|=1\right\}$ and $K_{c}(1)=\left\{L\in K(1):\left\|u\right\|=1,v=0\right\}$. We define L = (**u**, **v**)for $L\in K_{a}(1)$, then

$$\text{L}=\underset{{\text{A}}_{L}}{\underset{︸}{\left(\frac{u}{\left\|u\right\|},\;\frac{v}{\left\|v\right\|}\right)}}\underset{{\text{B}}_{L}}{\underset{︸}{\left(\begin{array}{cc} \left\|u\right\| & 0\\ 0 & \left\|v\right\| \end{array}\right)}}$$ (71)

Thus, from the following mappings:

$${\text{A}}_{L}\to \underset{{\text{R}}_{\text{L}}}{\underset{︸}{\left(\frac{u}{\left\|u\right\|},\;\frac{v}{\left\|v\right\|},\;\frac{u\times v}{\left\|u\times v\right\|}\right)}}\in SO(3)$$ (72)

$${\text{B}}_{L}\to \underset{{\text{W}}_{L}}{\underset{︸}{\left(\begin{array}{cc} \left\|u\right\| & -\left\|v\right\|\\ \left\|v\right\| & \left\|u\right\| \end{array}\right)}}\in SO(2)$$ (73)

we obtain the mapping $\phi :K_{a}(1)\mapsto SO(3)\times SO(2)$ by [4]:

$$\phi (L)=({\text{R}}_{L},\;{\text{W}}_{L})$$ (74)

and it is called the orthogonal representation of $L\in K_{a}(1)$.

The above mapping fails for $K_{b}(1)$ and $K_{c}(1)$. In order to obtain a complete mapping from K(1) into SO(3)×SO(2), we add definition for $K_{b}(1)$ and $K_{c}(1)$ as follows:

$$\phi (L)=\{\begin{array}{l} \left(2vv^{\text{T}}-{\text{I}}_{3},\;{\text{W}}_{\pi /2}\right),\;L\in K_{b}(1)\\ \left(2uu^{\text{T}}-{\text{I}}_{3},\;{\text{I}}_{2}),\;L\in K_{c}(1\right) \end{array}$$ (75)

where W_π/2_ is the 2D rotation of angle *π*/2. An explanation of this definition will be given later.

Using the angular distance on SO(3)×SO(2), the following distance on K(1) is obtained:

$$d_{\text{O}}(L,\;L^{\prime })=d_{∠}(\phi (L),\;\phi (L^{\prime })),\;L,\;L^{\prime }\in K(1)$$ (76)

Since L∈K(1) if and only if **L** is the normalized Plücker coordinates of a 3D line L∈L(3); and ±L∈K(1) are the normalized Plücker coordinates of the same 3D line, the distance *d*_O_ leads directly to the following distance on 3D line space L(3):

$$d_{\text{O}}(L,\;L^{\prime })=\text{min}\left\{d_{∠}(\phi (L),\;\phi (L^{\prime })),\;d_{∠}(\phi (L),\;\phi (-L^{\prime }))\right\},\;L,\;L^{\prime }\in L(3)$$ (77)

and it is called the orthogonal distance of 3D lines.

Now, we can give an explanation for the definition Eq (75). If $L,L^{\prime }\in K_{b}(1)$, then

$$\begin{array}{l} d_{\text{O}}(L,\;L^{\prime })=\text{arccos}\left(2{\left(v^{\text{T}}v^{\prime }\right)}^{2}-1\right)\\ \;\;\;\;\;\;\;\;\;\;\;\;\;\;\;\;=2\cdot \theta (v\text{,}v^{\prime }) \end{array}$$ (78)

Thus, the first mapping in the definition Eq (75) is meant the orthogonal distance of two lines passing through the origin is just twice their included angle.

Similarly, If $L,L^{\prime }\in K_{c}(1)$, then $d_{\text{O}}(L,\;L^{\prime })=2\cdot \theta (u\text{,}u^{\prime })$. Since **u** and **u** ^**′**^ are the normalized coordinates of the infinite lines L and $L^{\prime }$, respectively, and they are the normal vectors of plane passing through L and that passing through $L^{\prime }$. Hence, the second mapping in the definition Eq (75) is meant the orthogonal distance of two infinite lines L and $L^{\prime }$ is just twice the included angle of the two planes.

### 5.2 Quasi-Riemannian Metric on 3D Line Space

Based on the Riemannian metric [16] and analysis in Appendix II, the quasi-Riemannian distance on K(1) leads directly to the quasi-Riemannian distance on L(3):

$$d_{\text{QR}}(L,\;L^{\prime })=\text{min}\left\{d_{\text{K}}(L,\;L^{\prime }),d_{\text{K}}(L,\;-L^{\prime })\right\}$$ (79)

It is not difficult to verify that: lines L and $L^{\prime }$ are coplanar if and only if their Plücker coordinates satisfy **L** ^T^K**L** ^**′**^ = 0. Thus, the quasi-Riemannian distance of coplanar lines is given by the following formula:

$$d_{\text{QR}}(L,\;L^{\prime })=\text{min}\left\{\text{arccos}(L^{\text{T}}L^{\prime }),\;\pi -\text{arccos}(L^{\text{T}}L^{\prime })\right\}$$ (80)

### 5.3 Comparison of the Three Metrics

**Fig 3:**
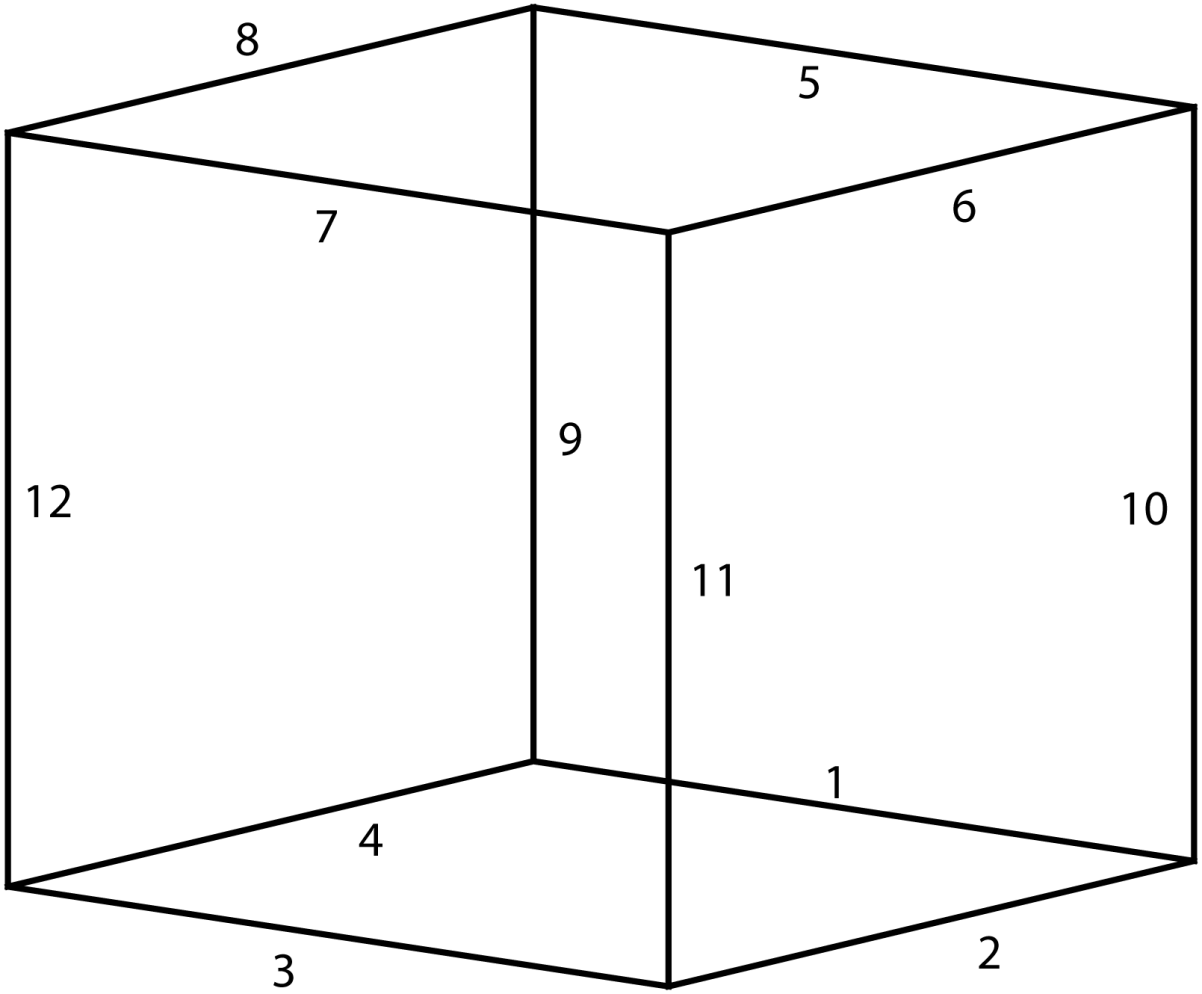
12 edges on the unit cube. (a) Euclidean metric; (b) Orthogonal metric; (c) Quasi-Riemannian metric.

**Fig 4:**
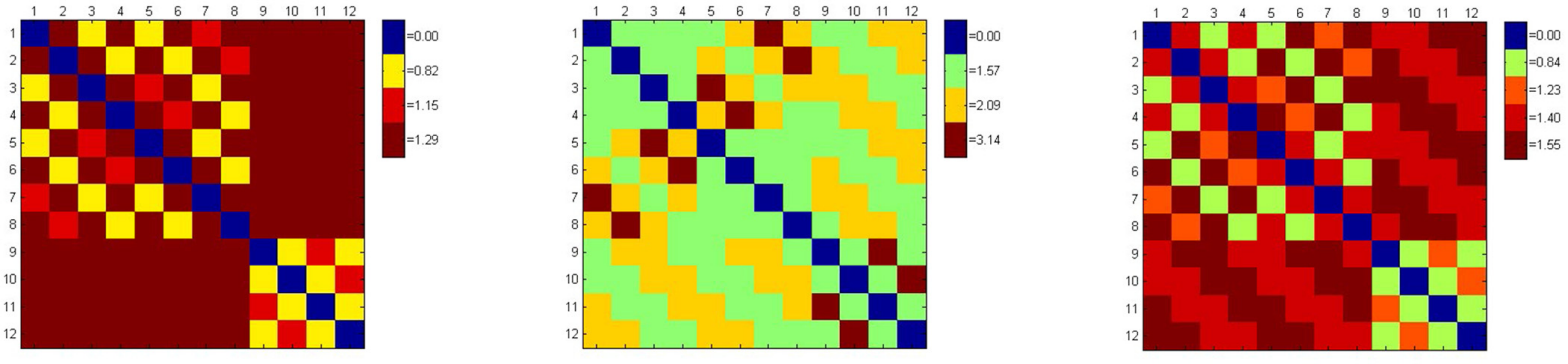
Distances between the edges on the unit cube by the three metrics.

In order to compare the performance of different metrics, we gerenated a 3D unit cube centered at the origin in space, and Fig 3 shows the 12 edges of the unit cube. Fig 4(a), 4(b) and 4(c) shows respectively the distances between the edges computed by the Euclidean metric, the Orthogonal metric, and the quasi-Riemannian metric, where different distance values are represented with different colors.

Based on their relative positions, the edge pairs belong to either the two parallel relationships (P-I and P-II) or the two orthogonal relationships (O-I and O-II) are listed as below:

$$\begin{array}{l} \text{P-I}=\left\{\left(\text{1,3}\right),\left(\text{1,5}\right),\left(\text{2,4}\right),\left(\text{2,6}\right),\left(\text{3,7}\right),\left(\text{4,8}\right),\left(\text{5,7}\right),\left(\text{6,8}\right),\left(\text{9,10}\right),\left(\text{9,12}\right),\left(\text{10,11}\right),\left(\text{11,12}\right)\right\}\\ \text{P-II}=\left\{\left(\text{1,7}\right),\left(\text{2,8}\right),\left(\text{3,5}\right),\left(\text{4,6}\right),\left(\text{9,11}\right),\left(\text{10,12}\right)\right\}\\ \text{O-I}=\left\{\begin{array}{l} \left(\text{1,2}\right),\left(\text{1,4}\right),\left(\text{1,9}\right),\left(\text{1,10}\right),\left(\text{2,3}\right),\left(\text{2,10}\right),\left(\text{2,11}\right),\left(\text{3,4}\right),\left(\text{3,11}\right),\left(\text{3,12}\right),\left(\text{4,9}\right),\left(\text{4,12}\right),\\ \left(\text{5,6}\right),\left(\text{5,8}\right),\left(\text{5,9}\right),\left(\text{5,10}\right),\left(\text{6,7}\right),\left(\text{6,10}\right),\left(\text{6,11}\right),\left(\text{7,8}\right),\left(\text{7,11}\right),\left(\text{7,12}\right),\left(\text{8,9}\right),\left(\text{8,12}\right) \end{array}\right\}\\ \text{O-II}=\left\{\begin{array}{l} \left(\text{1,6}\right),\left(\text{1,8}\right),\left(\text{1,11}\right),\left(\text{1,12}\right),\left(\text{2,5}\right),\left(\text{2,7}\right),\left(\text{2,9}\right),\left(\text{2,12}\right),\left(\text{3,6}\right),\left(\text{3,8}\right),\left(\text{3,9}\right),\left(\text{3,10}\right),\\ \left(\text{4,5}\right),\left(\text{4,7}\right),\left(\text{4,10}\right),\left(\text{4,11}\right),\left(\text{5,11}\right),\left(\text{5,12}\right),\left(\text{6,9}\right),\left(\text{6,12}\right),\left(\text{7,9}\right),\left(\text{7,10}\right),\left(\text{8,10}\right),\left(\text{8,11}\right) \end{array}\right\} \end{array}$$

Each of the three metrics can give a unique distance for each relationship, as shown in Table 4. However, from Table 4 it can be seen that the Euclidean metric could not distinguish between O-I and O-II; the orthogonal metric could not distinguish between P-I and O-I; while the quasi-Riemannian metric gives different distances for all four relationships, and these distances are consistent with our intuition that the distances for P-I, P-II, O-I and O-II should increase gradually. This observation implies that the quasi-Riemannian metric is reasonable than the Euclidean metric or the orthogonal metric.

**Table 4:** Distances computed by the three metrics for P-I, P-II, O-I and O-II.

	P-I	P-II	O-I	O-II
*d*_E_	0.82	1.15	1.29	1.29
*d*_O_	1.57	3.14	1.57	2.09
*d*_QR_	0.84	1.23	1.40	1.55

In the experiments of this paper, the quasi-Riemannian metric is used to evaluate the 3D errors of line triangulations. In real experiment, the true line and the estimated line are close with each other, so they can be considered as lying on the same plane. Therefore, the Quasi-Riemannian metric would be $d_{\text{QR}}(L,\;L^{\prime })=\text{arccos}(L^{\text{T}}L^{\prime })$. Let arccos(**L** ^T^ **L** ^**′**^) = *θ*. Since the angle of the two lines is small, so the Euclidean metric ($\left\|L\text{-}L^{\prime }\right\|=\sqrt{2-2L^{\text{T}}L^{\prime }}=\sqrt{2-2\text{cos}\theta }=2\text{sin}\left(\theta /2\right)$) can be approximated by 2sin(*θ*/2)≈*θ*. Therefore, the Quasi- Riemannian metric is equal to the Euclidean metric. The same situation also applies to the Orthogonal metric. As a result, the three metrics would be equal to each other or equal up to a scale factor.

## Experiments with Simulated Data

**Fig 5:**
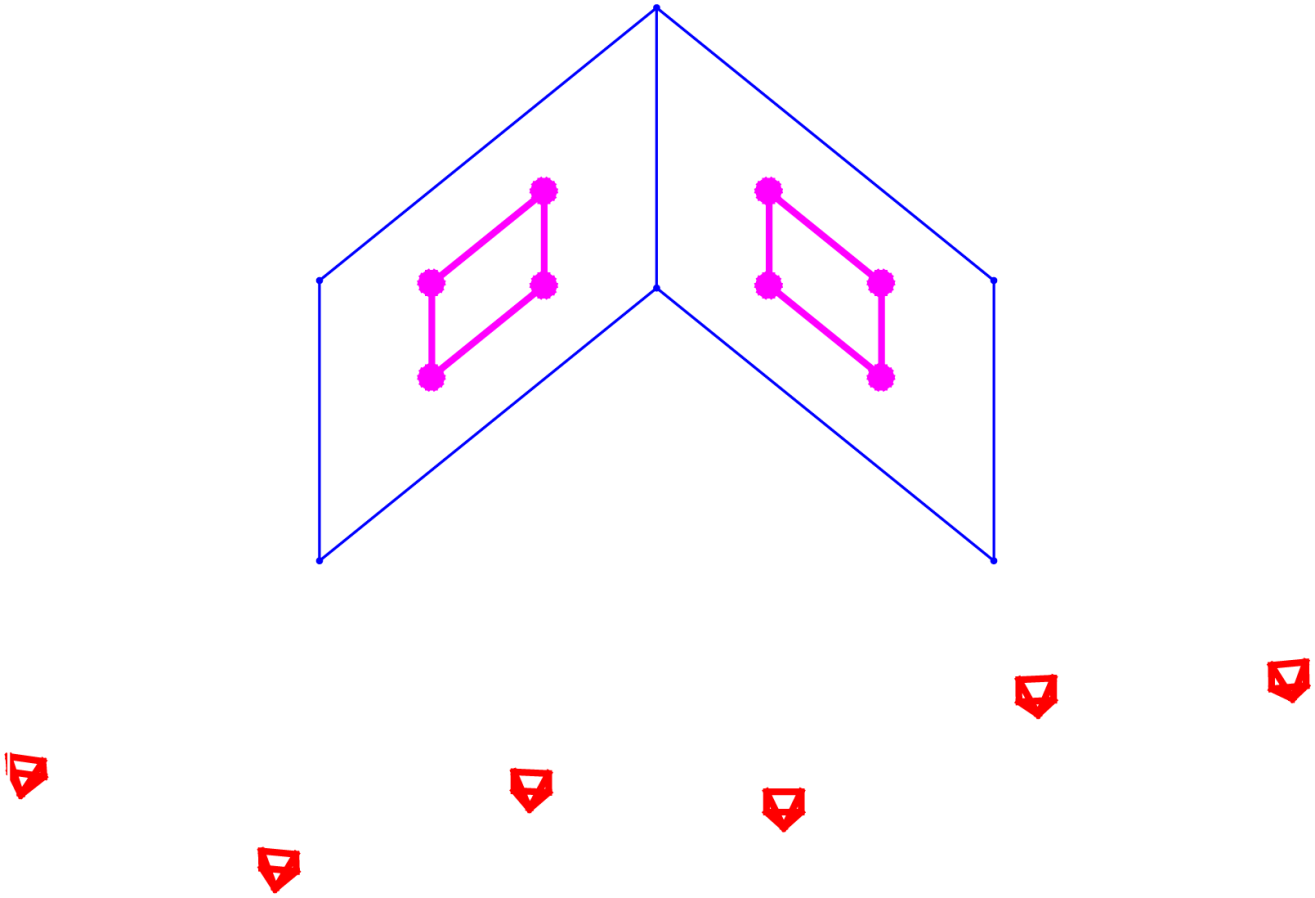
Eight 3D lines (in pink color) on two orthogonal planes used in the simulations, where the small pyramids stand for camera viewpoints.

In the experiments of this section, we simulated eight 3D space lines on two orthogonal planes, as shown in Fig 5. Using the synthetic data, we generated six images by adjusting the cameras location and parameters. The size of the images is of 1024×1024. In order to simulate the effect of image noise, we evenly sample 20 points on each image line segment, and add Gaussian noise with zero mean and *σ* standard deviation to these sampled image points, then, the actual projected image line is fitted by the orthogonal least squares fitting from these noise-corrupted point set.

We evaluated and compared the performance of the linear algorithm LIN [4], the proposed linear algorithm LINa; and the optimal algorithms based on the algebraic optimality criterion (AOC): OPTa-I and OPTa-II. The used criteria of evaluation are RMS (root mean square) of the 3D errors (i.e., the quasi-Riemannian distance of reconstructed line to its ground truth), the algebra errors, and the orthogonal errors.

### 6.1 Stability to Noise

**Fig 6:**
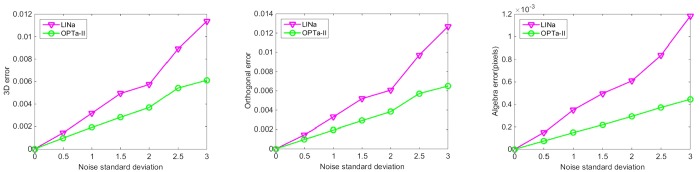
Stability of the algorithms with respect to different noise levels. (a) 3D errors; (b) algebraic errors; (c) orthogonal errors.

This experiment is to test the numerical stability of the algorithms with respect to different noise levels in the same geometric configuration. During the experiment, Gaussian noise with zero mean and *σ* standard deviation is added to each image point, and the noise level *σ* varies from 0.0 to 3.0 pixels in steps of 0.5, and 150 independent trials are carried out under each noise level. Fig 6 shows the experimental results on 6 views.

According to Lemma 1, LIN and LINa algorithm should yield the same result. On the other hand, since OPTa-I and OPTa-II algorithms both solve the algebraic-error minimization problem with the same error cost function, the two optimization algorithms should yield comparable estimation results, and only difference may be caused by the computational errors when solving the high-degree functions. These results have been verified by the experiments. In our experiments, both the LIN and the LINa algorithms produce the same errors, while the OPTa-I and the OPTa-II yield very close results, thus, we only show the results of LINa and OPTa-II in Fig 6. From this experiment, we can see that the RMS errors of all the algorithms increase with the increase of noise levels. The two optimal algorithms based on the AOC yield lower 3D errors, algebraic errors, and orthogonal errors than the two linear algorithms. Please note that since the three criteria are with different meanings and units, they are not comparable to each other.

In the experiments, both the OPTa-I and OPTa-II algorithms rarely have the situation of no real solutions. With the increase of noise level and image number, the possibility of no real solutions will increase slowly.

We also compared the computational cost of these algorithms. The real computation time of the LIN, LINa, OPTa-I, and OPTa-II algorithms are 0.002, 0.002, 11.681, 36.688 seconds, respectively. The two linear algorithms have comparable running time, while the two optimal algorithms are much computational intensive. Among the two optimal algorithms, the OPTa-I is faster than the OPTa-II since the former only needs to solve a 2-degree polynomial equation system, while the OPTa-II needs to solve one 6-degree and one 10-degree systems. Thus, OPTa-I is a better choice in practice.

### 6.2 Stability to Configurations

**Fig 7:**
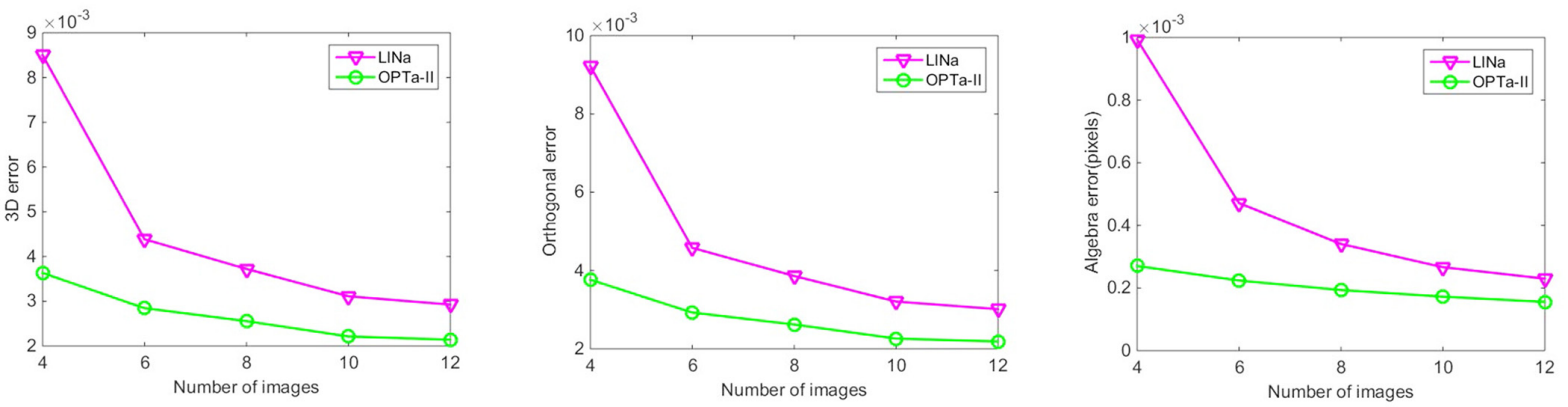
Stability of different algorithms with respect to geometrical configurations. (a) 3D errors; (b) Algebraic errors; (c) orthogonal errors.

This experiment is to test the numerical stability of the algorithms with respect to geometrical configurations. The number of views varies from 4 to 12 in steps of 2 during the experiments. At each number of views, 150 independent trials are carried out. Fig 7 shows the experimental results at noise level *σ* = 1.5, where only the results from LINa and OPT-II are plotted, as analyzed in Section 6.1, the LIN and LINa algorithms yield the same results, and the OPTa-I and OPTa-II produce very similar results. We can see from this experiment that the RMS error of all the algorithms decreases when the number of view increases. The two optimal algorithms outperform the two linear algorithms in term of 3D error, algebraic error, and orthogonal error.

## Experiments with Real Images

**Fig 8:**
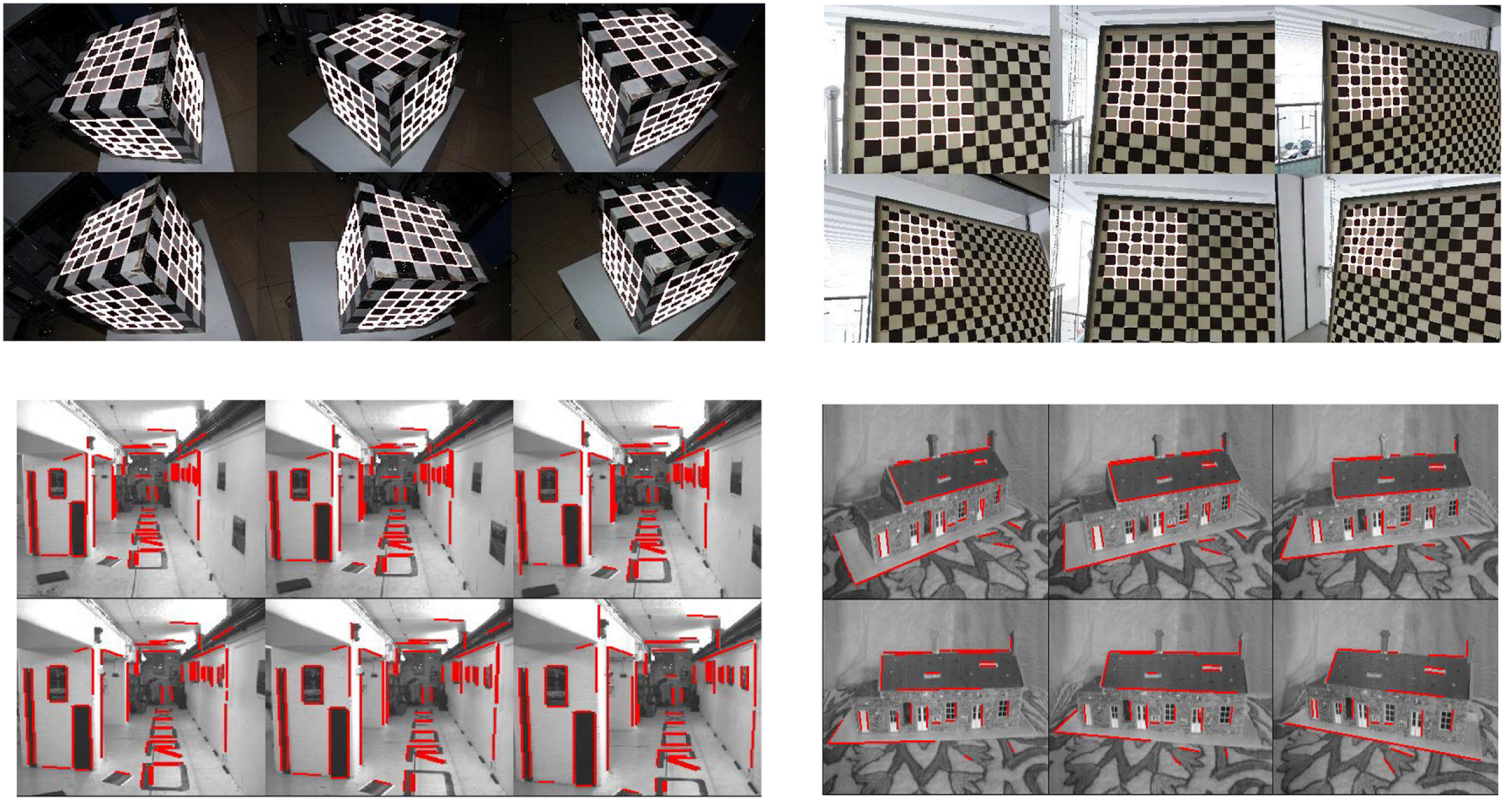
Image sets used in the experiments. (a) Calibration cube; (b) planar checkerboard; (c) Corridor; (d) Model House.

The proposed algorithms were evaluated using extensive real images. The experimental results on four data sets are reported below. As shown in Fig 8, the used images include a calibration cube, a planar checkerboard, and the Oxford datasets “model house” and “corridor” (http://www.robots.ox.ac.uk/~vgg/data/data-mview.html). The lines marked with white and red in these images are used to test the algorithms.

For the calibration cube, six images were taken by a Nikon D40 camera, with the image size of 3008×2000. The correspondences between the 3D points on the cube and their images are used to compute the camera matrices. For the planar checkerboard, six images were taken by a Sony HX5C camera, with the image size of 2592×1944, while the camera matrices are computed by the calibration toolbox (http://www.vision.caltech.edu/bouguetj/calib_doc/). For the model house images and the corridor images, the camera matrices and the two end coordinates of the image lines are provided by the Oxford datasets.

**Fig 9:**
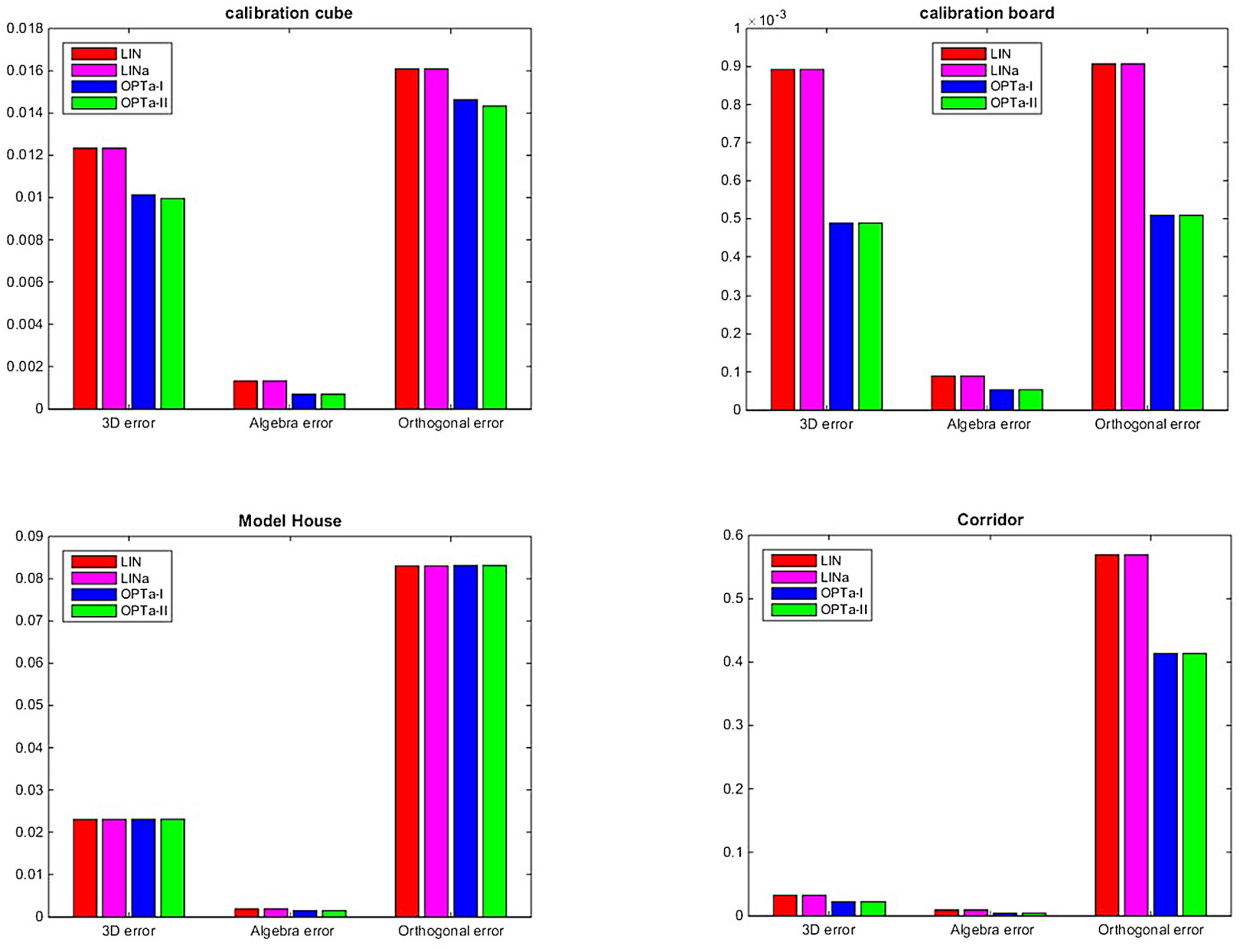
Experimental results. (a) Calibration cube; (b) planar checkerboard; (c) model house; (d) corridor.

**Fig 10:**
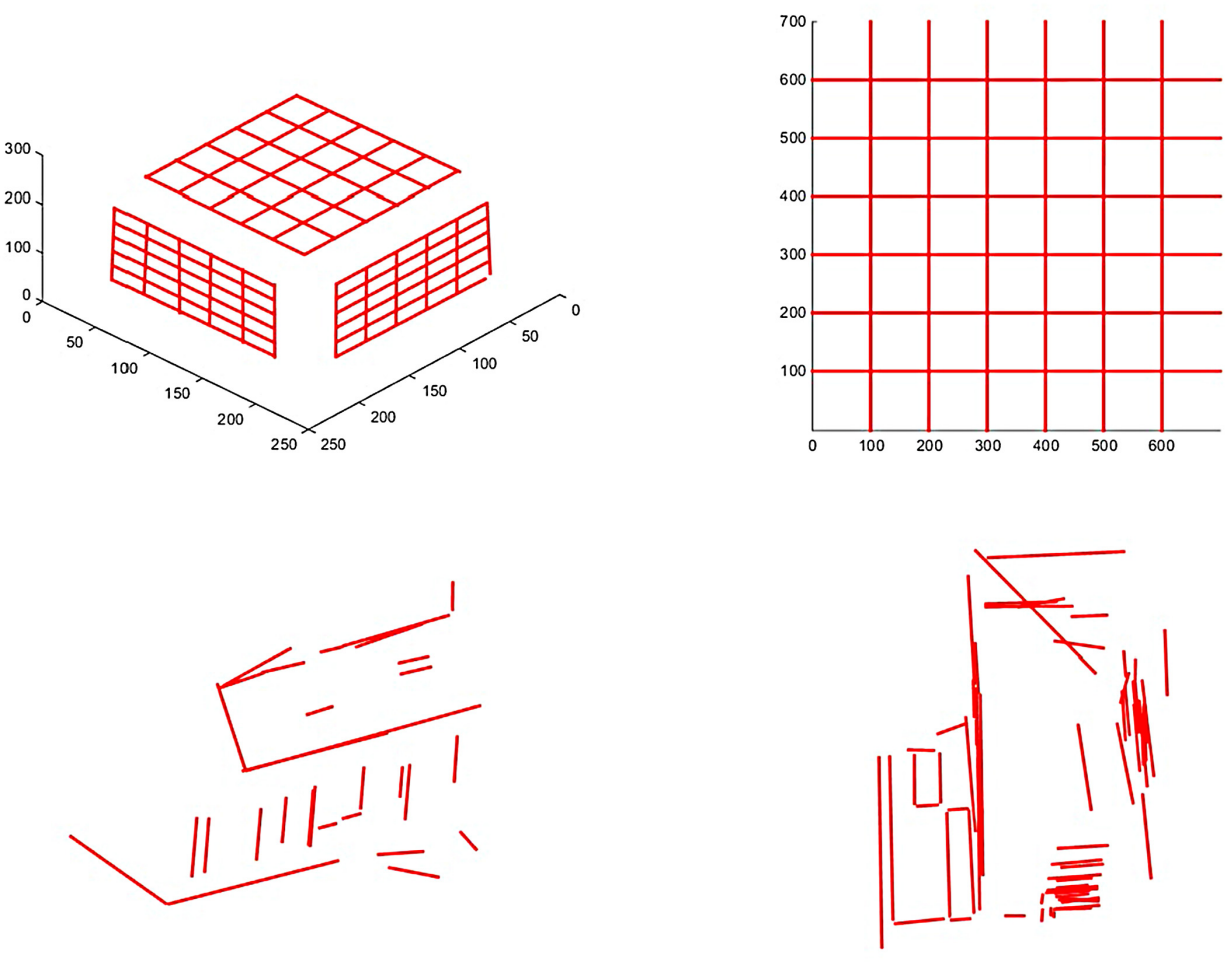
3D Reconstruction results. (a) Calibration cube; (b) planar checkerboard; (c) model house; (d) corridor.

Fig 9 shows the 3D errors, algebra errors, and orthogonal errors of different algorithms associated with the four data sets. From these experiments we can obtain the same conclusion as the simulation tests. The two optimal algorithms yield similar results which are better than those from the two linear algorithms. Although we plot the 3D error, algebraic error, and orthogonal error in one graph in Fig 9, these three errors are not comparable to each other since they are obtained using different criteria with different units. Fig 10 shows the 3D reconstruction results of the fours objects using the OPTa-I algorithm. The 3D models of these lines are correctly recovered by the proposed algorithm.

## Conclusion

In this paper, we have investigated line triangulations and line metrics. First, a new formula for the Plücker correction is introduced, by which a new linear algorithm for line triangulation is proposed. Then, two optimal algorithms are proposed from the algebraic optimality criterion. In addition, two metrics in 3D line space, the orthogonal metric and the quasi-Riemannian metric, are proposed for the quality evaluation of line triangulations. The experiments using simulated data and real images validate the proposed algorithms and show that the optimal solution can reconstruct more accurate 3D lines.

## Appendix I: Angular Metric on SO(3)×SO(2)

Let $SO(3)=\left\{\text{R}\in R^{3\times 3}:{\text{RR}}^{\text{T}}=\text{I},\text{ det}(\text{R})=1\right\}$ be the 3D rotation group. For R∈SO(3) there is the following angle- axis representation:

$$\text{R}=\text{I}+\text{sin}(\theta ){\left[a\right]}_{\times }+(1-\text{cos}(\theta )){\left[a\right]}_{\times }^{2}$$ (81)

where *θ* (0≤*θ*≤*π*) and **a**(‖**a**‖ = 1) are respectively the rotation angle and rotation axis of R, and the rotation angle satisfies:

$$\theta =\text{arccos}\left(\frac{\text{tr}(\text{R})-1}{2}\right)$$ (82)

The angular distance of $\text{R, }{\text{R}}^{\prime }\in SO(3)$ is defined as [8]

$$d_{∠}^{\left(3\right)}(\text{R},\;{\text{R}}^{\prime })=\text{arccos}\left(\frac{\text{tr}({\text{R}{\text{R}}^{\prime }}^{\text{T}})-1}{2}\right)$$ (83)

Similarly, for the 2D rotation group $SO(2)≜\left\{\text{W}\in R^{2\times 2}:{\text{WW}}^{\text{T}}=\text{I},\text{ det}(\text{W})=1\right\}$, the angular distance is defined as

$$d_{∠}^{\left(2\right)}(\text{W},\;{\text{W}}^{\prime })=\text{arccos}\left(\frac{\text{tr}({\text{W}{\text{W}}^{\prime }}^{\text{T}})}{2}\right)$$ (84)

According to the angular distances $d_{∠}^{\left(3\right)}$ and $d_{∠}^{\left(2\right)}$, the angular distance on SO(3)×SO(2) can be defined as

$$\begin{array}{l} d_{∠}^{}(\text{X},\;{\text{X}}^{\prime })=d_{∠}^{\left(3\right)}(\text{R},\;{\text{R}}^{\prime })+d_{∠}^{\left(2\right)}(\text{W},\;{\text{W}}^{\prime }),\\ \text{ X}=(\text{R},\text{ W}),\;{\text{X}}^{\prime }=({\text{R}}^{\prime },\;{\text{W}}^{\prime })\in SO(3)\times SO(2) \end{array}$$ (85)

Since the geodesic distances of metric spaces $\left(SO(3),\;d_{∠}^{\left(3\right)}\right)$ and $\left(SO(2),\;d_{∠}^{\left(2\right)}\right)$ are the angular distances $d_{∠}^{\left(3\right)}$ and $d_{∠}^{\left(2\right)}$ themselves [8], it is not difficult to verify that:*d*_∠_ is also the geodesic distance of metric space $\text{(}SO(3)\times SO(2),\;d_{∠}^{})$.

## Appendix II: Quasi-Riemannian Metric on K(1)

We first state briefly the Riemannian metric on $S^{5}(1)$ in order to introduce quasi-Riemannian metric on K(1). Let *S* = (0,…,0,−1)^T^ and *N* = (0,…,0,1)^T^, called respectively the south pole and north pole of $S^{5}(1)$, we define the mappings $\varphi_{\pm }:U_{\pm }\to R^{5}$ as follows:

$$Y=\varphi_{\pm }(X)≜\frac{1}{1\pm x_{6}}\left(x_{1},x_{2}\ldots ,x_{5}\right),\;X\in U_{\pm }$$ (86)

where $U_{+}=S^{5}(1)\backslash \{S\}$ and $U_{-}=S^{5}(1)\backslash \{N\}$. Their inverse mappings are

$$X=\varphi_{\pm }^{-1}(Y)=\frac{1}{1+\sum_{i}y_{i}^{2}}{\left(2y_{1},\ldots ,2y_{5},\pm \left(1-\sum_{i}y_{i}^{2}\right)\right)}^{\text{T}},\;Y\in \varphi (U_{\pm })$$ (87)

and $J=\left\{\left(U_{+},\varphi_{+}),(U_{-},\varphi_{-}\right)\right\}$ is a smooth structure on $S^{5}(1)$. The Riemannian metric on $S^{5}(1)$ induced by the standard Euclidean metric, $h=\sum_{i}{\left(dx_{i}\right)}^{2}$, in $R^{6}$ is

$$g=\frac{4}{{\left(1+\Sigma_{i}y_{i}^{2}\right)}^{2}}\sum_{i}{\left(dy_{i}\right)}^{2}$$ (88)

Let $\gamma =\left\{Y(t)={\left(y_{1}(t),\ldots ,y_{n}(t)\right)}^{\text{T}}:0\leq t\leq 1\right\}$ be a smooth or piecewise smooth curve in $S^{5}(1)$, its length is defined as

$$L(\gamma )=\int_{0}^{1}\sqrt{\frac{4}{{\left(1+\Sigma_{i}y_{i}^{2}\left(t\right)\right)}^{2}}\sum_{i}{\left(dy_{i}(t)/dt\right)}^{2}}dt=\int_{0}^{1}\frac{2}{1+{\left\|Y(t)\right\|}^{2}}\left\|\frac{dY(t)}{dt}\right\|\;dt$$ (89)

For $Y_{0},\;Y_{1}\in S^{5}(1)$, let $\Gamma_{Y_{0},\;Y_{1}}$ be the set of all smooth or piecewise smooth curves with the endpoints at **Y**_0_ and **Y**_1_, the Riemannian distance induced by the metric Eq (88) is

$$d_{\text{S}}(Y_{0},\;Y_{1})≜\text{inf}\left\{L(\gamma ):\gamma \in \Gamma_{Y_{0},\;Y_{1}}\right\}$$

$=L(\varepsilon \text{(}Y_{0},\;Y_{1}\text{)})\text{ where}\varepsilon \text{(}Y_{0},\;Y_{1}\text{)}$ is the short arc from **Y**_0_ to **Y**_1_ on a great circle in $S^{5}(1)$.

$$=\text{arccos}(Y_{0}^{\text{T}}Y_{1})$$ (90)

It is not difficult to verify that the Riemannian distance *d*_s_ and the Euclidean distance *d*_E_ (= ‖**Y**_0_−**Y**_1_‖) both satisfy the following relation:

**Lemma 2:** For $Y_{0},Y_{1},Y_{2},Y_{3}\in S^{5}(1)$,

$$d_{\text{S}}(Y_{0},\;Y_{1})<d_{\text{S}}(Y_{2},\;Y_{3})\;\Leftrightarrow \;d_{\text{E}}(Y_{0},\;Y_{1})<d_{\text{E}}(Y_{2},\;Y_{3})$$ (91)

Next, we introduce the quasi-Riemannian distance on K(1) from the Riemannian metric on $S^{5}(1)$.

For $\;X_{0}=\left(\begin{array}{c} u_{0}\\ v_{0} \end{array}\right),\;X_{1}=\left(\begin{array}{c} u_{1}\\ v_{1} \end{array}\right)\in K(1)$, let

$$\begin{array}{l} X(t)=(1-t)X_{0}+tX_{1}\\ \;\;\;\;\;\;\;\;=\left(\begin{array}{c} (1-t)u_{0}+tu_{1}\\ (1-t)v_{0}+tv_{1} \end{array}\right)≜\left(\begin{array}{c} u(t)\\ v(t) \end{array}\right),\;0\leq t\leq 1 \end{array}$$ (92)

Then, we have the following lemma.

**Lemma 3:** (a) If **u**_0_±**v**_0_≠−(**u**_1_±**v**_1_), then **u**(*t*)±**v**(*t*)≠0, *t*∈[0,1]

(b) If **u**_0_+**v**_0_ = −(**u**_1_+**v**_1_), then

u(t)+v(t)≠0,  t∈[0,1]\{1/2};  u(1/2)+v(1/2)=0

(c) If **u**_0_−**v**_0_ = −(**u**_1_−**v**_1_), then

u(t)−v(t)≠0,  t∈[0,1]\{1/2};  u(1/2)−v(1/2)=0

**Proof:** From **u**(*t*)±**v**(*t*) = (1−*t*)(**u**_0_±**v**_0_)+*t*(**u**_1_±**v**_1_),

$$u(t)\pm v(t)=0\;\Leftrightarrow \;\{\begin{array}{l} u_{0}\pm v_{0}=-s(u_{1}\pm v_{1})\\ s=t/(1-t) \end{array}$$ (93)

Since ‖**u**_0_±**v**_0_‖ = ‖**u**_1_±**v**_1_‖ = 1, *s* = 1 by the first equation in Eq (93), thus **u**_0_±**v**_0_ = −(**u**_1_±**v**_1_). Therefore (a) holds. If **u**_0_±**v**_0_ = −(**u**_1_±**v**_1_), there must be *t* = 1/2 by the second equation in Eq (93), and thus (b) holds. Similarly, (c) holds.

Clearly, the short arc from **X**_0_ to **X**_1_ on a great circle in $S^{5}(1)$ is

$$\bar{X}(t)=\frac{X(t)}{\left\|X(t)\right\|}=\frac{1}{\left\|X(t)\right\|}\left(\begin{array}{c} u(t)\\ v(t) \end{array}\right),\;0\leq t\leq 1$$ (94)

Since

$$\begin{array}{lll} X^{\text{T}}(t)\text{K}X(t) & = & 2u^{\text{T}}(t)v(t)\\ & = & 2t(1-t)\left(u_{0}^{\text{T}}v_{1}+v_{0}^{\text{T}}u_{1}\right)\\ & = & 2t(1-t)X_{0}^{\text{T}}\text{K}X_{1},\;\forall 0\leq t\leq 1 \end{array}$$

we have

- If $X_{0}^{\text{T}}\text{K}X_{1}=0$, then $\bar{X}(t)\in K(1)\text{ for }0\leq t\leq 1$;
- If $X_{0}^{\text{T}}\text{K}X_{1}\neq 0$, then $\bar{X}(t)\notin K(1)\text{ for }0<t<1$.

For the case (a), the Riemannian distance on $S^{5}(1)$ leads directly to the Riemannian distance between **X**_0_ and **X**_1_ in K(1):

$$d_{K}(X_{0},\;X_{1})=\text{arccos}(X_{0}^{\text{T}}X_{1})$$ (95)

We consider the case (b) next. According to Proposition 1 and Lemma 3, the best approximation of $\bar{X}(t)\;\left(t\neq 1/2\right)$ on the sub-manifold K(1) under the Euclidean metric is

$$X^{*}(t)=\frac{1}{2}\left(\begin{array}{c} \frac{u(t)+v(t)}{\left\|u(t)+v(t)\right\|}+\frac{u(t)-v(t)}{\left\|u(t)-v(t)\right\|}\\ \frac{u(t)+v(t)}{\left\|u(t)+v(t)\right\|}-\frac{u(t)-v(t)}{\left\|u(t)-v(t)\right\|} \end{array}\right)\in K(1)$$ (96)

By Lemma 2, X*(*t*) is also the best approximation of $\bar{X}(t)$ on K(1) under the Riemannian metric, thus X*(*t*) is the orthogonal projection of $\bar{X}(t)$ on K(1) under the Riemannian metric. By Lemma 3, X*(*t*) is a smooth or piecewise smooth curve on K(1). Thus by letting $Y^{*}(t)=\varphi \left(X^{*}(t)\right)$, a quasi-Riemannian distance between **X**_0_ and **X**_1_ in K(1) is obtained using the Riemannian metric on $S^{5}(1)$:

$$d_{\text{K}}(X_{0},\;X_{1})=\int_{0}^{1}\underset{f(t)}{\underset{︸}{\frac{2}{1+{\left\|Y^{*}(t)\right\|}^{2}}\left\|\frac{dY^{*}(t)}{dt}\right\|}}\;dt$$ (97a)

**Proposition 3:** The integration Eq (97a) can be expressed as:

$$d_{\text{K}}(X_{0},\;X_{1})=\sqrt{2}\int_{0}^{1/2}{\left(\frac{a}{{\left(t^{2}+a\right)}^{2}}+\frac{b}{{\left(t^{2}+b\right)}^{2}}\right)}^{1/2}\;dt$$ (97)

where,

$$\begin{array}{l} a=\{\begin{array}{l} (2-q_{+})/(4q_{+}),\;q_{+}\neq 0\\ 0,\;q_{+}=0 \end{array}\\ b=\{\begin{array}{l} (2-q_{-})/(4q_{-}),\;q_{-}\neq 0\\ 0,\;q_{-}=0 \end{array}q_{\pm }=1-(X_{0}^{\text{T}}X_{1}\pm X_{0}^{\text{T}}\text{K}X_{1}), \end{array}$$ (98)

Specifically, if $X_{0}^{\text{T}}\text{K}X_{1}=0$ then $d_{\text{K}}(X_{0},\;X_{1})=\text{arccos}(X_{0}^{\text{T}}X_{1})$, i.e., Eq (91) is a special case of Eq (97).

**Proof:** By some mathematical manipulation, the integrand of Eq (97a) can be expressed as:

$$\begin{array}{l} f(t)=\frac{1}{\sqrt{2}}{\left(\frac{(2-q_{+})q_{+}}{{\left(2q_{+}(t^{2}-t)+1\right)}^{2}}+\frac{(2-q_{-})q_{-}}{{\left(2q_{-}(t^{2}-t)+1\right)}^{2}}\right)}^{1/2}\\ \;\;\;\;\;\;\;\;=\frac{1}{\sqrt{2}}{\left(\frac{(2-q_{+})q_{+}}{{\left(4q_{+}{\left(t-1/2\right)}^{2}+(2-q_{+})\right)}^{2}}+\frac{(2-q_{-})q_{-}}{{\left(4q_{-}{\left(t-1/2\right)}^{2}+(2-q_{-})\right)}^{2}}\right)}^{1/2}\\ \;\;\;\;\;\;\;\;=\frac{1}{\sqrt{2}}{\left(\frac{a}{{\left({\left(t-1/2\right)}^{2}+a\right)}^{2}}+\frac{b}{{\left({\left(t-1/2\right)}^{2}+b\right)}^{2}}\right)}^{1/2} \end{array}$$ (99)

Thus,

$$\begin{array}{l} d_{\text{K}}(X_{0},\;X_{1})=\frac{1}{\sqrt{2}}\int_{0}^{1}{\left(\frac{a}{{\left({\left(t-1/2\right)}^{2}+a\right)}^{2}}+\frac{b}{{\left({\left(t-1/2\right)}^{2}+b\right)}^{2}}\right)}^{1/2}\;dt\\ \;\;\;\;\;\;\;\;\;\;\;\;\;\;\;\;\;\;\;=\sqrt{2}\int_{0}^{1/2}{\left(\frac{a}{{\left(t^{2}+a\right)}^{2}}+\frac{b}{{\left(t^{2}+b\right)}^{2}}\right)}^{1/2}\;dt \end{array}$$ (100)

If $X_{0}^{\text{T}}\text{K}X_{1}=0$, then $a=b=4^{-1}(1+X_{0}^{\text{T}}X_{1})/(1-X_{0}^{\text{T}}X_{1})$, and

$$\begin{array}{l} d_{\text{K}}(X_{0},\;X_{1})=2\sqrt{a}\int_{0}^{\frac{1}{2}}\frac{1}{t^{2}+a}\;dt=2\text{arctan}\frac{1}{2\sqrt{a}}\\ \;\;\;\;\;\;\;\;\;\;\;\;\;\;\;\;\;\;=2\text{arctan}\sqrt{\frac{1-X_{0}^{\text{T}}X_{1}}{1+X_{0}^{\text{T}}X_{1}}}=\text{arccos}(X_{0}^{\text{T}}X_{1}) \end{array}$$ (101)
